# *Neisseria meningitidis porA*, *fetA* and *fHbp* gene distribution in Western Australia 2000 to 2011

**DOI:** 10.1186/s12879-014-0686-x

**Published:** 2014-12-12

**Authors:** Peter Boan, Norhaliza Metasan, Simone Tempone, Gerry Harnett, David J Speers, Anthony D Keil

**Affiliations:** Department of Microbiology, PathWest Laboratory Medicine WA, Princess Margaret Hospital for Children, Roberts Road, Subiaco, 6008 Australia; School of Pathology and Laboratory Medicine, University of Western Australia, Crawley, WA Australia; Department of Microbiology, PathWest Laboratory Medicine WA, Queen Elizabeth II Medical Centre, Nedlands, WA Australia; School of Medicine and Pharmacology, University of Western Australia, Crawley, WA Australia; Department of Microbiology and Infectious Diseases, PathWest Laboratory Medicine WA, Royal Perth Hospital, Wellington Street, Perth, 6000 WA Australia

**Keywords:** Neisseria meningitidis, Factor H binding protein, PorA, FetA, Meningococcal epidemiology, Meningococcal B vaccine

## Abstract

**Background:**

*PorA*, *fetA* and *fHbp* are three antigen encoding genes useful for meningococcal typing and FHbp is an important component of meningococcal B vaccines.

**Methods:**

We performed sequence analysis of meningococcal *porA*, *fetA* and *fHbp* genes on 128 isolates from Western Australia, relating results to age, gender, race and geographic region.

**Results:**

We found predominantly PorA subtypes P1.22,14-16 (n = 23) and P1.7-2,4 (n = 19); FetA subtypes F1-5 (n = 41), F3-6 (n = 11), F5-1 (n = 10), F5-2 (n = 9), F5-5 (n = 8), F3-3 (n = 8); and FHbp variant groups 1 (n = 65) and 2 (n = 44). PorA P1.22,14-16 and FHbp variant group 2 were associated with younger age and aboriginality.

**Conclusions:**

FHbp modular groups of the bivalent recombinant FHbp vaccine and the multicomponent 4CMenB vaccine make up 8.3% and 47.7% respectively of the examined serogroup B isolates from 2000–2011, however to estimate vaccine efficacy requires an account of all vaccine antigens and their levels of expression.

**Electronic supplementary material:**

The online version of this article (doi:10.1186/s12879-014-0686-x) contains supplementary material, which is available to authorized users.

## Background

Invasive meningococcal infection is a rapidly progressing disease with a case fatality rate of 10% and 10-20% of survivors experience serious permanent sequelae. Of the 12 *Neisseria meningitidis* serogroups, the majority of invasive disease is caused by serogroups A, B, C, Y and W. Serogroup B, C and Y are responsible for most infections in North America and Europe, while serogroup A continues to cause major epidemics in sub-Saharan Africa [1]. Western Australia is a highly multicultural population of 2.4 million people living in an area greater than 2.5 million square kilometres encompassing tropical and temperate climatic zones, factors which would predict meningococcal strain variability. However the level of urbanisation should be appreciated with 78% of the population residing within the greater area of the capital city, Perth [2]. For informing public health responses, meningococcal typing is performed by sequencing antigen encoding genes such as *factor H binding protein* (*fHbp*), variable regions of *porA* and *fetA*, and by multilocus sequence typing (MLST). The seven housekeeping genes analysed by MLST are under stabilising selection making the technique useful for tracking long-term national and international epidemiology [3]. PorA is a porin that is a major constituent of the outer membrane of most meningococcal isolates and contains two major variable regions (VR1 and VR2), designated in short form as P1.VR1,VR2. Currently there are 249 unique peptide sequences grouped into 10 families reported for VR1, with 694 unique peptide sequences grouped into 20 VR2 families [4]. FetA is an iron-regulated meningococcal outer membrane protein, with 417 unique variable region peptides categorised into nine families, F1 to F9 [4]. Factor H is a human factor that inhibits the alternative complement pathway which, when bound to the meningococcal cell surface by the Factor H binding protein (FHbp), leads to impaired human complement activation and reduced microbial killing [5]. The classification of FHbp is complicated by multiple overlapping nomenclature schemes. Fletcher *et al.* [6] classified FHbp into subfamilies A and B and Masignani *et al.* [7] into variant groups 1 (corresponding to subfamily B), 2 and 3 (together corresponding to subfamily A). More recently, modular groups I-IX have been assigned based on different combinations of either variant group 1 or 3 peptide types in five modular segments (A, B, C, D, E). Brehony *et al.* [8] proposed an alternative nomenclature scheme assigning a number to each distinct *fHbp* allele or peptide. The numbers are arbitrary being assigned sequentially to new alleles/peptides.

The development of a broadly protective meningococcal B vaccine is proving a formidable challenge. The meningococcal B polysaccharide is an autoantigen expressed by a number of host tissues and, as such is not a vaccine candidate due to poor immunogenicity. The detergent-extracted outer membrane vesicle (OMV) vaccines are most active against certain strains because the serum bactericidal antibody (SBA) responses are largely directed against PorA protein, which is antigenically highly variable and poorly cross reactive [9]. Likewise the vaccine potential of FetA has been limited by antigenic variability [10]. In contrast, broad serogroup B meningococcal immunity is possible with recombinant FHbp vaccines as antibodies generated against FHbp subfamily A strains have cross immunity to other subfamily A types, and antibodies against FHbp subfamily B strains have cross immunity to other subfamily B types [9],[11]. The bivalent recombinant vaccine manufactured by Pfizer containing FHbp subfamily A and B variants (subfamily A strain is variant group 3, Brehony peptide 45, and subfamily B strain is variant group 1, Brehony peptide 55) is well tolerated in adolescents and toddlers and induces robust SBA responses against a selection of serogroup B strains [12],[13]. The second major meningococcal B vaccine (4CMenB) manufactured by Novartis contains an FHbp subfamily B variant (variant group 1, peptide 1) in combination with the meningococcal antigens neisserial adhesin A (NadA), neisserial heparin-binding antigen (NHBA), and an OMV from the New Zealand outbreak strain NZ98/254. It also appears to be well tolerated and immunogenic against the serogroup B strains tested [14],[15] and was registered by the Therapeutic Goods of Australia 14 August 2013, but predicted efficacy of the vaccines will depend on knowledge of local circulating FHbp, NadA, NHBA, and PorA types and the expression of these antigens [16],[17]. This study was therefore undertaken to describe the distribution of meningoccocal PorA, FetA and FHbp types in Western Australia and provide information to help predict the likelihood of meningococcal B vaccine efficacy in our region.

## Methods

### Selection of *Neisseria meningitidis*isolates

Between 1 January 2000 and 31 December 2011, 497 cases of invasive meningococcal disease (mandatory notification is required in Western Australia) were notified to the Communicable Disease Control Directorate (CDCD), the central public health unit of Western Australia. We tested 128 isolates of the 271 cases which had a stored meningococcal culture isolate. A confirmed invasive meningococcal case requires isolation of *N. meningitidis* or detection of specific meningococcal DNA sequences by nucleic acid amplification testing from a normally sterile site; or clinical evidence of disease (an illness deemed compatible with meningococcal disease by the treating physician) with suggestive laboratory evidence such Gram negative diplococci seen on microscopy from a sterile site. Also notified are probable cases defined by a clinically compatible illness including haemorrhagic rash or a close contact with a confirmed case within the previous 60 days, with no evidence for other causes of the clinical condition [18]. The statewide policy is to send a subculture from all invasive meningococcal cases to the state meningococcal laboratory at the Princess Margaret Hospital for Children (PMH). Isolates were not available for all notified cases due to culture negative meningococcal diagnoses by nucleic acid detection (125 cases), strain loss and solely clinical diagnoses. The 128 meningococcal isolates examined were those isolates stored only at PMH rather than additionally at the meningococcal culture collection of the large Western Australian public laboratory at the Queen Elizabeth II Medical Centre. The number of isolates (percentage of notified cases) tested from each year is as follows; 2000: 21 (24.4%), 2001: 24 (32.4%), 2002: 13 (19.4%), 2003: 10 (21.7%), 2004: 13 (32.5%), 2005: 12 (25.5%), 2006: 6 (28.6%), 2007: 3 (15.0%), 2008: 9 (37.5%), 2009: 12 (42.8%), 2010: 1 (4.5%), 2011: 4 (18.2%). In addition to the demographic data of age, sex and geographic region of residence, we recorded aboriginality, survival and the anatomical site of the isolate. Isolates were serogrouped with Remel monovalent agglutinating sera (Thermo Scientific Remel, Lenexa, KA, USA).

### Polymerase chain reaction (PCR) and sequencing

Isolates were stored at −80°C in tryptic soy broth containing 15% glycerol. After thawing and culture, several colonies on chocolate blood agar were placed in 10% saline and heated to 70°C for one hour exposing the DNA. The nested PCR primers of Table 1 were designed using GenBank sequences [19], to target conserved regions of the *porA*, *fetA* and *fHbp* genes (accession numbers in Additional file 1: Table S5). Faint or no bands on gel electrophoresis were obtained with single step PCR techniques leading to the use of nested PCR reactions. We chose to design primers believing they may perform superiorly to some published primers based on homology to a greater proportion of GenBank sequences. Because at initial planning the chosen portion of the *fetA* gene looked too long for a single sequence, we amplified two overlapping gene segments (overlap not in the variable region), and we needed to use an alternate reverse primer to obtain a complete *fHbp* sequence for six isolates (J42, K25, K41, K113, K276, P601-further details regarding these isolates are found Additional file 1: Table S6). Sequencing primers were the same as second round primers.

**Table 1:** **Primers for** ***porA*** **,** ***fetA*** **and** ***fHbp*** **PCR reactions**

Primer	Sequence (5′-3′)
*porA* 1^st^ round forward primer	5′-AAA CTT ACC GCC CTC GTA-3′
*porA* 1^st^ round reverse primer	5′-TTA GAA TTT GTG GCG CAA ACC GAC-3′
*porA* 2^nd^ round forward primer^a^	5′-CCG CAC TGC CGC TTG CGG-3′
*porA* 2^nd^ round reverse primer^a^	5′-CGC ATA TTT AAA GGC ATA-3′
*fetA* (segment 1) 1^st^ round forward primer^b^	5′-CGG CGC MAG CGT ATT CGG-3′
*fetA* (segment 1) 1^st^ round reverse primer^c^	5′-GCG GTT TGA TTT CCT GAT GG-3′
*fetA* (segment 1) 2^nd^ round forward primer^a^	5′-TTR TTC TCT TAC AAC CGC ARC-3′
*fetA* (segment 1) 2^nd^ round reverse primer^a,c^	5′-GCG GTT TGA TTT CCT GAT GG-3′
*fetA* (segment 2) 1^st^ round forward primer	5′-GCC TTG CCG AAC AAA CYC-3′
*fetA* (segment 2) 1^st^ round reverse primer	5′-CGC CCA ATT CGT AAC CGT-3′
*fet A* (segment 2) 2^nd^ round forward primer^a^	5′-TCA ACT ACC GCC ATC AGG A-3′
*fetA* (segment 2) 2^nd^ round reverse primer^a^	5′-TTT GAT GGT YTG CCA RAA GTA GC-3′
*fHbp* 1^st^ round foward primer	5′-GTG AAY CGA ACT RCC TTC TRY-3′
*fHbp* 1^st^ and 2^nd^ round reverse primer^a^	5′-TGT TCR ATT TTG CCG TRY-3′
	5′-TGG TIT TTT TYA TCC GGC TTR-3′
*fHbp* 2^nd^ round forward primer^a^	5′-CCC TGA TTC TGA CCG CCT G-3′

^a^Second round primers also used as sequencing primers.

^b^S1 primer from Thompson *et al.* [12].

^c^S4 primer from Thompson *et al.* [12].

First round PCR reactions (20 μL total volume) comprised 8 μL of sample and 12 μL of mix containing 0.2 μmol/L of each deoxynucleotide triphosphate (dNTP), 1.5 mmol/L of MgCl_2_, 0.5 units of AmpliTaq Gold DNA polymerase (Applied Biosystems, Foster City, CA, USA), 0.2 μmol/L of both forward and reverse primer, 0.01% bovine serum albumin, 2 μL of buffer PE (Qiagen, Valencia, CA, USA) and 8.424 μL of H_2_O. PCR was conducted with an initial 10 minutes at 95°C, followed by 45 cycles of denaturation at 94°C for 30 seconds, annealing at 55°C for 30 seconds, and extension at 72°C for 60 seconds; after which there was 1 cycle of 72°C for 7 minutes, then holding at 4°C. Second round PCR reactions (20.5 μL total volume) comprised 0.5 μL of 1^st^ round PCR product and 20 μL of mix containing 0.2 μmol/L for each dNTP, 2 mmol/L MgCl_2_, 0.5 units of AmpliTaq Gold DNA polymerase, 0.2 μmol/L of both forward and reverse primer, 0.01% bovine serum albumin, 2.04 μL of buffer PE (Qiagen) and 15.946 μL of H_2_O. We performed PCR with the same conditions as the 1^st^ round PCR.

Second round product was detected by 2.5% ethidium bromide gel electrophoresis and clean up of this product was accomplished with Exo SAP-IT (USB Corporation, Cleveland, OH, USA).

Sequencing reactions (total volume 10 μL) comprised 2.5 μL of cleaned 2^nd^ round PCR product, and 7.5 μL of mix containing 1 μL of Big Dye Terminator 3.1 reaction mix (Applied Biosystems), 1.5 μL of Big Dye Terminator 5x buffer, 3.2 μL (0.43 μmol/L) primer and 1.8 μL H_2_O. We performed 45 cycles of denaturation at 95°C for 10 seconds and annealing/extension at 60°C for 30 seconds, followed by holding at 4°C. Sequencing product was centrifuged into 20 μL of formamide via DyeEx 2.0 spin kit filters (Qiagen, Valencia, CA, USA) and analysed in the ABI 3130xI DNA sequencer (Applied Biosystems). We sequenced in the forward and reverse direction to improve accuracy. All PCR and sequencing reactions were performed using ABI 2720 thermocyclers (Applied Biosystems).

### Bioinformatic analysis

Sequences were examined in BioEdit version 7.1.3 [20] and were aligned by CLUSTAL W [21]. PorA, FetA and FHbp types were determined by importing nucleotide sequences at the Neisseria Sequence Typing home page [4]. Our sequences contained FHbp modular segments A, B, C, D akin to many prior FHbp studies. Lacking modular segment E meant FHbp allele identification were “closest hits”. Continuous variables were examined by Mann Whitney *U* test and categorical variables by Chi-squared test or Fisher’s exact test as appropriate. Statistics including logistic and multiple regression were performed in MedCalc version 12.4.0.0. A two-sided p value of <0.05 was used to indicate statistical significance.

### Ethical considerations

We characterised isolates submitted as a component of the mandatory reporting of invasive meningococcal disease cases according to the Western Australian Health Act [22]. Human Research Ethics Committee review was not required as the research is of negligible risk and conforms to section 5.1.18-5.1.21 of the Australian National Statement on Ethical Conduct in Human Research [23].

## Results

### Study population

We examined isolates from 128 cases with a mean age of 4.6 years (range 0.09 to 90.8 years) and 74 were male and 54 female. Twenty (15.9% of 126 cases where race was known) were aboriginal comparing to 3.8% aboriginality of the Western Australian population [24]. There were 104 cases from metropolitan Perth, 20 from regional Western Australia, and one each from interstate and overseas (2 cases from unknown region). Isolates were predominately of serogroup B (n = 109) and C (n = 15), with only 2 isolates each of serogroup W and Y. Isolate serogroup by year was; 2000: 17 B (85.0%), 3 C (15.0%), 2001: 20 B (83%), 3 C (12.5%), 1 W (4.2%), 2002: 10 B (71.4%), 4 C (28.6%), 2003: 11 B (100%), 2004: 9 B (75.0%), 2 C (16.7%), 1 W (8.3%), 2005: 12 B (100%), 2006: 5 B (83.3%), 1 C (16.7%), 2007: 3 B (100%), 2008: 9 B (100%), 2009: 9 B (75.0%), 2 C (16.7%), 1 Y (8.3%), 2010: 1 B (100%), 2011: 3 B (75.0%), 1 Y (25.0%).

We analysed how representative the 128 selected cases were of all 497 reported invasive meningococcal cases over the time period. A similar proportion of the examined 128 isolates compared to the 497 cases reported to the public health department were male (57.8% vs 58.7%, p = 0.927), aboriginal (16.5% vs 15.9%, p = 1.00) and serogroup B (85.1% vs 87.1%, p = 0.521), however the selected cases compared to the total cases were significantly younger (0–4 years old 51.6% vs 41.3%, p = 0.045) and urbanised (84.4% vs 72%, p = 0.006).

### Genotyping results

A *porA*, *fetA* and *fHbp* sequence was obtained for all isolates and while most could be identified (Additional file 1: Table S6), 32 *fetA* and 3 *fHbp* translated sequences (Additional file 1: Table S7) were treated as no result which contained a stop codon. PorA P1.22,14-16 (n = 23) and P1.7-2,4 (n = 19) were the predominant PorA types. The main FetA types were F1-5 (n = 22), F3-6 (n = 11), F5-1 (n = 10), F5-2 (n = 9), F5-5 (n = 8) and F3-3 (n = 8). The predominant FHbp alleles were 654 (n = 28), 733 (n = 17) and 15 (n = 12). FHbp variant group 1 was most common (n = 65), followed by group 2 (n = 44) and group 3 (n = 15), meaning a similar number of subfamily A (n = 60, being variant group 2 and 3) and B (n = 65, being variant group 1).

### Genotyping association with other variables

Using the main PorA, FetA and FHbp subtypes we examined for association with patient demographics (Tables 2, 3 and 4). PorA subtype P1.22,14-16 and FHbp variant group 2 were both significantly associated with younger age and a higher proportion of aboriginal cases. A multivariate logistic regression model of aboriginality which incorporated P1.22,14-16, FHbp variant group 2 and age, demonstrated FHbp was independently associated with aboriginality (p = 0.027, regression coefficient 0.164, standard error 0.073). A logistic regression model of age incorporating P1.22, 14–16, variant group 2 and aboriginality showed none of these variables were independently associated with age (p = 0.293, p = 0.052, p = 0.821 respectively).

**Table 2:** **Demographic factors related to predominant PorA VR1, VR2 subtypes**

	P1.22,14-16	Non P1.22,14-16	p value	P1.7-2,4	Non P1.7-2,4	p value
**Age** ^*^	1.32	6.01	<0.005^†^	6.29	4.33	<0.005^†^
**Male**	12 (52.2)	62 (59.0)	0.710	14 (73.7)	60 (55.0)	0.205
**Aboriginal**	9 (39.1)	11 (10.5)	0.002	0 (0.0)	20 (18.5)	0.089
**Regional**	7 (30.4)	13 (12.4)	0.065	6 (31.6)	14 (12.8)	0.08
**TOTAL**	23	105		19	109	

Values are numbers (%). ^*^Geometric mean age in years. ^†^p value based on arithmetic means.

**Table 3:** **Demographic factors related to predominant FetA subtypes**

	F1-5	Non F1-5	p value	F3-6	Non F3-6	p value	F5-1	Non F5-1	p value
**Age** ^*^	3.65	4.78	0.500^†^	8.39	4.33	0.255^†^	5.46	4.51	0.866^†^
**Male**	11 (50.0)	63 (59.4)	0.563	5 (45.0)	64 (54.7)	0.786	8 (80.0)	64 (54.2)	0.942
**Aboriginal**	5 (22.7)	15 (14.1)	0.505	0 (0.0)	20 (17.1)	0.290	1 (10.0)	19 (16.1)	0.955
**Regional**	7 (31.8)	13 (12.3)	0.048	0 (0.0)	20 (17.1)	0.290	0 (0.0)	20 (17.0)	0.355
**TOTAL**	22	106		11	117		10	118	

Values are numbers (%). ^*^Geometric mean age in years. ^†^p value based on arithmetic means.

**Table 4:** **Demographic factors related to FHbp variant groups**

	VG1	Non VG1	p value	VG2	Non VG2	p value	VG3	Non VG3	p value
**Age** ^*^	6.06	3.12	0.027^†^	2.43	6.15	0.006^†^	6.57	4.17	0.429^†^
**Male**	39 (60.0)	32 (53.3)	0.960	25 (55.5)	44 (55.0)	0.994	9 (60.0)	60 (54.5)	0.851
**Aboriginal**	5 (7.7)	15 (23.1)	0.017	14 (31.1)	6 (7.5)	0.001	1 (6.7)	19 (17.3)	0.500
**Regional**	6 (9.2)	14 (23.3)	0.0343	11 (24.4)	9 (11.2)	0.093	3 (20.0)	17 (15.5)	0.940
**TOTAL**	65	60		45	80		15	110	

Values are numbers (%). ^*^Geometric mean age in years. ^†^p value based on arithmetic means.

The PorA, FetA and FHbp subtypes were evenly represented through 2000 to 2009 (data not shown). Too few isolates from 2010 and 2011 were tested to comment on trends in these latter years. The number of cases was highest in the third quarter (n = 45) most closely associated with the winter season, compared to second quarter (n = 31), fourth quarter (n = 23) and first quarter (n = 23). Demographic and genotypic data for each isolate are presented in Additional file 1: Table S6.

## Discussion

Since the 1980s invasive meningococcal disease in Australia has occurred in a hypersporadic pattern with a current rate (2011) of 0.9 cases per 100,000 population, and the last reported outbreak in Western Australia occurred prior to 1995 [25],[26]. There is scant Australian published data regarding meningococcal genotypes. The Australian National Neisseria Network collecting data on invasive meningococcal disease from each state reference laboratory, have only published PorA typing results of serogroup B isolates from 2010 and 2011, showing the predominant types of P1.7-2,4, P1.7,16-26 and P1.22,14 [26],[27]. Overall P1.22,14-16 and P1.7-2,4 were the predominant subtypes in our study (we had 5 of P1.7,16-26 and 7 of P1.22,14) though we examined very few isolates from 2010 and 2011 making the comparison difficult. Our PorA typing results suggest the most prevalent MeNZB OMV strain (P1.7-2,4) in isolation may provide poor coverage for our local strains, as this subtype comprised only 15.6% of our meningococcal B isolates. We found PorA P1.22,14-16 and FHbp variant group 2 were associated with younger age and a higher proportion of aboriginal cases. A multivariate logistic regression model of aboriginality which incorporated P1.22,14-16, FHbp variant group 2 and age, demonstrated FHbp variant group 2 to be independently associated with aboriginal cases. However, FHbp being an imperfect marker of lineage means the observed association could be due to other characteristics of a lineage in aboriginal people rather than FHbp itself. Considering meningococcal prevention in the high risk aboriginal population, FHbp subfamily B of the multicomponent Novartis meningococcal B vaccine made up only 26.3% (5/19) of serogroup B aboriginal cases. However, the significance of this finding is unclear as we have not tested for the other components of the vaccine or gene expression [9].

Consistent with most countries examined apart from South Africa, we found the percentage of meningococcal strains carrying FHbp subfamily B variants (53.1%) was higher than those carrying FHbp subfamily A variants [28],[29]. We found 2 isolates (both serogroup B) precisely matching the subfamily A and no isolates precisely matching the subfamily B components of the bivalent Pfizer vaccine [12]. Regarding this vaccine’s modular and variant groups, we found 5 modular group V and 4 modular group IV serogroup B isolates (8.26% of serogroup B isolates), 57 variant group 1 and 14 variant group 3 serogroup B isolates (65% of serogroup B isolates). There were no isolates precisely matching the subfamily B FHbp component of the multicomponent 4CMenB Novartis meningococcal B vaccine [30], however there were 52 modular group I and 57 variant group 1 serogroup B isolates (47.7% and 52.3% of serogroup B isolates respectively). If vaccine-induced anti-FHbp antibodies have highest bactericidal activity against isolates from a modular group matching the vaccine strain(s) [31], our data would suggest that the multicomponent Novartis vaccine may provide better overall coverage of our strains. Determination of the relative frequencies of NadA and NHBA types are also needed to predict the multicomponent vaccine efficacy, however it should be noted that genetic data alone has insufficient discriminatory power to estimate vaccine coverage. Further information would be provided by testing our isolates with the recently described meningococcal antigen typing system (MATS), a method accounting not only for gene presence but also for the level of antigen gene expression and cross reactivity, which can be used to predict strain responsiveness to a vaccine [16],[17]. Nissen *et al.* tested 373 Australian invasive meningococcal B isolates (including an unreported percentage of isolates from Western Australia) from 2009–2011 by MATS with regard to the multicomponent Novartis meningococcal B vaccine, finding 76% of isolates demonstrated relative potency over the positive bacterial threshold for at least one antigen vaccine component [32].

Our study looks at three relevant meningococcal genes, including a key component of meningococcal B vaccines. It would be interesting to compare our results to similar data from other parts of Australia and our region as the antigenic variability we have demonstrated in Western Australia may be peculiar to our diverse geographical, climatic and cultural landscape. There is potential for selection bias as we excluded isolates that were also stored at the Queen Elizabeth II Medical Centre public laboratory, which although being a site that receives isolates from a large number of laboratories statewide, is likely biased towards strains from adult cases by servicing a large adult tertiary hospital. Indeed our set were significantly younger and less likely to reside rurally compared to all reported cases. Only five isolates from 2010/2011 were examined leaving us unable to draw conclusions regarding these years and the interpretation of temporal trends was hampered by the variable percentage of isolates examined per year (range 4.5%-42.8%). Despite attempts to optimise primer design, we acknowledge there are inefficiencies in our assays suggested by the requirement of a nested approach and for some isolates alternate *fHbp* primers. It may have been more prudent to utilise published *porA*, *fetA* and *fHbp* primers. Additionally, although a longer *fetA* sequence was obtained, the *fetA* variable region is within our second PCR segment allowing determination of *fetA* characterisation from this single PCR and sequence. Finally, we recognise that MLST testing would have allowed more precise genetic information to compare to the worldwide meningococcal epidemiology, however was not feasible due to labour and time constraints.

## Conclusions

We sequenced *porA*, *fetA* and *fHbp* genes of 128 meningococcal isolates from Western Australia over the period 2000–2011. We found several types predominate in our population and there was an association of PorA P1.22,14-16 and FHbp variant group 2 with younger age and aboriginality. We found the FHbp modular groups of the bivalent Pfizer and multicomponent Novartis vaccines constitute 8.26% and 47.7% respectively of the examined serogroup B isolates from the 11 year period, though this supplies only a crude genotypic prediction of vaccine efficacy not taking into account all vaccine antigens or their levels of expression.

## Additional file

## Electronic supplementary material

Additional file 1: GenBank Accession numbers of isolates utilised in primer design, isolate demographic and genotyping data, and sequences with no match at the Neisseria Typing home page. (DOCX 43 KB)

## Abbreviations

CDCD: Communicable Diseases Control Directorate of Western Australia
FHbp: Factor H binding protein
MATS: Meningococcal antigen typing system
MLST: Multi-locus sequence typing
NadA: Neisserial adhesion A
NHBP: Neisserial heparin-binding protein
OMV: Outer membrane vesicle
SBA: Serum bactericidal antibody
VR: Variable region
