# Supplementary material for: Neisseria meningitidis porA, fetA and fHbp gene distribution in Western Australia 2000 to 2011
Source: BMC Infect Dis. 2014 Dec 12;14:686. doi: 10.1186/s12879-014-0686-x (PMC4266217; doi:10.1186/s12879-014-0686-x)
Supplement: Supplementary file 1 — Additional file 1: GenBank Accession numbers of isolates utilised in primer design, isolate demographic and genotyping data, and sequences with no match at the Neisseria Typing home page. (DOCX 43 KB) [file 12879_2014_686_MOESM1_ESM.docx]

**SUPPLEMENTARY DATA**

**Table 5. GenBank Accession numbers of isolates utilised in primer design.**

***porA***

[AF171638](http://www.ncbi.nlm.nih.gov/entrez/viewer.fcgi?db=nuccore&id=AF171638), [AF146084](http://www.ncbi.nlm.nih.gov/entrez/viewer.fcgi?db=nuccore&id=AF146084), [AF151677](http://www.ncbi.nlm.nih.gov/entrez/viewer.fcgi?db=nuccore&id=AF151677), [AF051542](http://www.ncbi.nlm.nih.gov/entrez/viewer.fcgi?db=nuccore&id=AF051542), [AJ012727](http://www.ncbi.nlm.nih.gov/entrez/viewer.fcgi?db=nuccore&id=AJ012727), [U92947](http://www.ncbi.nlm.nih.gov/entrez/viewer.fcgi?db=nuccore&id=U92947), [U92926](http://www.ncbi.nlm.nih.gov/entrez/viewer.fcgi?db=nuccore&id=U92926), [AF159360](http://www.ncbi.nlm.nih.gov/entrez/viewer.fcgi?db=nuccore&id=AF159360), [AF167347](http://www.ncbi.nlm.nih.gov/entrez/viewer.fcgi?db=nuccore&id=AF167347), [AF239810](http://www.ncbi.nlm.nih.gov/entrez/viewer.fcgi?db=nuccore&id=AF239810), [AF112453](http://www.ncbi.nlm.nih.gov/entrez/viewer.fcgi?db=nuccore&id=AF112453), [U92948](http://www.ncbi.nlm.nih.gov/entrez/viewer.fcgi?db=nuccore&id=U92948), [AF148643](http://www.ncbi.nlm.nih.gov/entrez/viewer.fcgi?db=nuccore&id=AF148643), [AF182278](http://www.ncbi.nlm.nih.gov/entrez/viewer.fcgi?db=nuccore&id=AF182278), [U93907](http://www.ncbi.nlm.nih.gov/entrez/viewer.fcgi?db=nuccore&id=U93907), [AF051541](http://www.ncbi.nlm.nih.gov/entrez/viewer.fcgi?db=nuccore&id=AF051541), [AJ012736](http://www.ncbi.nlm.nih.gov/entrez/viewer.fcgi?db=nuccore&id=AJ012736), [U93906](http://www.ncbi.nlm.nih.gov/entrez/viewer.fcgi?db=nuccore&id=U93906), [AF163831](http://www.ncbi.nlm.nih.gov/entrez/viewer.fcgi?db=nuccore&id=AF163831), [AF162345](http://www.ncbi.nlm.nih.gov/entrez/viewer.fcgi?db=nuccore&id=AF162345)

***fetA***

AF439260, AF439160, AF439183, AF439186, AF439187, AF439186, AF439193, AF439201, AF439212, AF439213, AF439214, AF439215, AF439217, AF439218, AF439234, AF439246, AF439260, AF439190, AF439159, AF439192, AF439229, U55377, AF439195, AF439256, AF439197, AF439176, AF439194, AF439196, AF439249, AF439247, U55378, U67313, U67314

***fHbp***

JF916579, AY548370, CP002419, JF916581, JF916582, JF916578, JF916579, CP002419, CP002422, CP002423, DQ324737, DQ324739, EU337063, EU541888, EU921901, JF916583, JF916582, AY548370, JF916579, CP002419, CP002422, CP002423, DQ324737, DQ324739, EU337063, EU541888, EU921901, EU5411897, JF916581, JG916582, FJ153808, FJ422921, FJ615424, FJ615426, FJ615429, FJ615431, FJ750978, FM999788, GQ219769, GQ219776, GQ405398, GU339518, GU339519, GU339524, GU339535, JF916577, JF916578

**Table 6. Isolate demographic and genotyping data.**

| **Assigned Code** | **Aboriginal** | **Regional** | **Year** | **Serogroup** | **PorA** | **FetA** | **FHbp allele** | **FHbp MG** | **FHbp VG** |
| --- | --- | --- | --- | --- | --- | --- | --- | --- | --- |
| **J107**  **J109**  **J147**  **J164**  **J19**  **J258**  **J291**  **J292**  **J312**  **J42**  **J52**  **K113**  **K172**  **K181**  **K236**  **K25**  **K274**  **K276**  **K294**  **K37**  **K41**  **K446**  **K60**  **L1**  **L416**  **L440**  **L466**  **L617**  **L658**  **M360**  **M46**  **M526**  **M603**  **N119**  **N262**  **N296**  **N299**  **N461**  **N648**  **P396**  **P450**  **P555**  **P583**  **P601**  **Q390**  **Q46**  **R371**  **R419**  **R46**  **S424**  **S442**  **S765**  **S788**  **T121**  **T233**  **T24**  **T435**  **T437**  **T53**  **T591**  **U630**  **W279**  **W283**  **K289**  **K324**  **L578**  **M235**  **J88**  **K288**  **N649**  **P105**  **P335**  **T505**  **W233**  **K447**  **M418**  **M492**  **N197**  **P381**  **N32**  **J156**  **K281**  **L223**  **L633**  **Q95**  **S419**  **S583**  **T218**  **T416**  **T639**  **W078**  **J142**  **J149**  **J171**  **J174**  **J202**  **J249**  **J41**  **K104**  **K163**  **K188**  **K263**  **K362**  **K373**  **L102**  **L106**  **L220**  **L265**  **L552**  **L635**  **M420**  **M441**  **M510**  **N266**  **N295**  **N321**  **P247**  **P297**  **P459**  **P641**  **Q203**  **Q234**  **Q49**  **R5**  **S504**  **S532**  **S697**  **T391** | N  N  N  N  N  N  N  N  N  N  Y  N  N  N  N  Y  N  U  N  N  Y  N  N  N  N  N  N  N  N  N  N  N  N  N  N  N  N  N  N  N  N  N  Y  N  N  N  N  N  N  N  Y  N  N  N  N  N  N  N  N  N  N  N  N  N  N  N  N  N  N  N  N  N  N  N  N  N  N  N  N  N  N  N  N  N  N  N  N  N  Y  N  N  N  N  N  N  Y  N  Y  N  Y  N  Y  N  N  N  N  Y  N  Y  N  N  Y  Y  N  N  Y  Y  N  N  Y  Y  N  Y  N  N  Y  U  N | N  N  Y  N  N  N  N  N  N  N  N  N  N  N  N  N  N  U  N  N  N  N  N  U  N  N  N  N  N  Y  N  N  N  N  N  N  N  N  N  N  N  N  N  N  N  Y  N  N  Y  N  N  N  N  N  N  N  N  Y  N  N  Y  N  N  N  N  N  Y  N  N  N  N  N  N  N  N  N  N  N  N  N  N  N  Y  N  N  N  N  Y  N  N  N  N  Y  N  N  N  N  N  N  N  N  N  N  N  N  N  Y  Y  Y  Y  N  Y  Y  N  Y  N  N  N  N  N  N  N  Y  Y  N  N  Y  N | 2000  2000  2000  2000  2000  2000  2000  2000  2000  2000  2000  2001  2001  2001  2001  2001  2001  2001  2001  2001  2001  2001  2001  2001  2002  2002  2002  2002  2002  2003  2003  2003  2003  2004  2004  2004  2004  2004  2004  2005  2005  2005  2005  2005  2006  2005  2007  2007  2007  2008  2008  2008  2008  2009  2009  2009  2009  2009  2009  2009  2010  2011  2011  2001  2001  2002  2003  2000  2001  2004  2004  2005  2009  2011  2001  2003  2003  2003  2005  2004  2000  2001  2002  2002  2006  2008  2008  2009  2009  2009  2011  2000  2000  2000  2000  2000  2000  2000  2001  2001  2001  2001  2001  2001  2002  2002  2002  2002  2002  2002  2003  2003  2003  2004  2004  2004  2005  2005  2005  2005  2006  2006  2006  2006  2008  2008  2008  2009 | B  B  B  C  C  B  B  B  B  C  B  B  C  B  C  B  B  B  C  B  B  B  B  B  C  C  B  B  B  B  B  B  B  B  B  B  C  B  B  B  B  B  B  B  B  B  B  B  B  B  B  B  B  C  B  C  B  B  B  B  B  B  B  B  B  B  B  B  B  B  C  B  Y  Y  B  B  B  B  B  B  B  B  C  B  B  B  B  B  B  B  B  B  B  B  B  B  B  B  B  B  B  W  B  B  B  B  B  B  B  C  B  B  B  B  W  B  B  B  B  B  B  B  B  C  B  B  B  B | P1.7-2,4  P1.7-2,4  P1.7-2,4  P1.5,2  P1.5-1,10-8  P1.5,2  P1.18,25  P1.19-1,26  P1.7,16-2  P1.18-1,3  P1.7-1,1  P1.7,16-26  P1.5-1,10-8  P1.7-2,4  P1.19-3,15  P1.19-1,26  P1.22,14-6  P1.7-2,4  P1.5-1,10-8  P1.7-2,4  P1.19,15  P1.7,16  P1.19-1,15  P1.7,16-2  P1.5-1,10-8  P1.5-1,10-1  P1.7-2,4  P1.5-2,10-11  P1.18-1,3  P1.7-2,4  P1.7,16-26  P1.22,14-6  P1.21-7,16  P1.5-2,10-11  P1.7,30-3  P1.7,16-26  P1.5-1,10-1  P1.7-2,4  P1.22,14  P1.5-2,10-11  P1.12-6,13-4  P1.19,15  P1.12-6,13-4  P1.7,30-3  P1.7-2,13-1  P1.7,16  P1.7-2,4  P1.5-2,10-11  P1.21-7,16-88  P1.7-2,4  P1.22,14-6  P1.19,4  P1.22,14  P1.5-1,10-8  P1.22,14  P1.5-1,10-8  P1.21,9  P1.5-1,10-4  P1.5-2,10-79  P1.22,14  P1.7-2,4  P1.22,14  P1.22,14  P1.7-2,4  P1.7-2,4  P1.7-2,4  P1.7-2,4  P1.7,30-3  P1.5-2,10-1  P1.17,16-3  P1.5-1,10-4  P1.5-2,10-1  P1.5-1,10-4  P1.5-11,2-2  P1.7-2,16  P1.19-1,15-11  P1.19-1,15-11  P1.5-2.10-11  P1.19-1,15-11  P1.22,14-6  P1.21,16  P1.18-1,3  P1.19-3,15  P1.7,16  P1.22,14  P1.18,25  P1.19-2,13-1  P1.7,16-26  P1.7-1,13-1  P1.17,16-3  P1.7,16-26  P1.22,14-6  P1.22,14-6  P1.19,15-1  P1.22,14-6  P1.7-1,1  P1.7-2,4  P1.21,14-16  P1.19-3,15  P1.22,14-6  P1.22,14-6  P1.18-1,3  P1.22,9  P1.21,16  P1.19,15  P1.22,14-6  P1.22,14-6  P1.22,14-6  P1.22,14-6  P1.7-2,4  P1.22,14-6  P1.22,14-6  P1.5-1,10-4  P1.22,14-6  P1.18-1,3  P1.22,14-6  P1.22,9  P1.22,14-6  P1.7-2,13-2  P1.22,14-6  P1.22,14-6  P1.5-1,10-1  P1.22,14-6  P1.7-2,4  P1.22,2  P1.31,16-55  P1.22,14-6  P1.22,14-6 | Nil  Nil  F1-5  F3-6  F3-6  F3-6  F5-5  F4-1  Nil  F3-6  F4-1  F1-19  F1-64  Nil  F5-5  F5-1  Nil  F1-5  F3-6  F1-5  F3-7  F3-3  F1-5  Nil  F3-6  F3-6  F5-18  Nil  Nil  F3-9  F3-3  Nil  F5-36  F5-2  F5-1  F3-3  F3-6  N  Nil  Nil  Nil  F5-1  Nil  F5-1  Nil  F3-3  F1-5  F5-2  F5-36  F1-5  F1-5  F5-1  F5-9  F5-8  Nil  F5-8  F5-12  F1-5  F5-2  F3-6  F1-5  F3-9  F3-9  F5-18  F5-18  F5-18  F1-5  F4-5  F5-2  F5-5  F3-6  F5-2  F1-15  Nil  F5-1  F5-1  F5-1  F5-2  F5-1  F1-5  F5-5  F3-6  Nil  F3-3  F5-5  F5-2  F3-9  F3-3  Nil  F5-5  F3-3  F1-5  F1-5  F5-5  F1-84  F4-1  Nil  F1-5  Nil  F1-5  F1-5  Nil  F5-12  F5-5  F5-1  F1-5  F5-24  Nil  Nil  F5-2  F1-5  F1-5  Nil  F1-5  F3-1  Nil  Nil  Nil  Nil  F5-9  Nil  F3-3  F1-5  F5-2  Nil  F3-9  Nil  F1-5 | 583  583  583  729  11  729  583  583  733  733  463  301  11  733  593  733  733  733  11  583  733  733  583  733  11  11  421  405  724  733  646  733  267  405  733  119  11  583  602  405  611  733  611  733  343  733  583  405  422  583  724  733  Nil  Nil  672  Nil  672  583  405  353  583  505  505  421  421  421  421  789  140  363  92  140  759  789  755  755  755  442  755  654  685  469  574  574  574  685  469  574  469  201  574  654  654  654  654  136  721  654  654  654  654  721  654  654  27  654  654  654  654  134  654  654  654  654  654  654  654  654  654  654  654  72  654  134  654  654  654  654 | I  I  I  I  I  I  I  I  I  I  I  I  I  I  I  I  I  I  I  I  I  I  I  I  I  I  I  I  I  I  I  I  I  I  I  I  I  I  I  I  I  I  I  I  I  I  I  I  I  I  I  I  Nil  Nil  I  Nil  I  I  I  I  I  I  I  II  II  II  II  III  III  III  III  III  III  III  IV  IV  IV  IV  IV  N  V  V  V  V  V  V  V  V  V  V  V  VI  VI  VI  VI  VI  VI  VI  VI  VI  VI  VI  VI  VI  VI  VI  VI  VI  VI  VI  VI  VI  VI  VI  VI  VI  VI  VI  VI  VI  VI  VI  VI  VI  VI  VI  VI  VI | 1  1  1  1  1  1  1  1  1  1  1  1  1  1  1  1  1  1  1  1  1  1  1  1  1  1  1  1  1  1  1  1  1  1  1  1  1  1  1  1  1  1  1  1  1  1  1  1  1  1  1  1  Nil  Nil  1  Nil  1  1  1  1  1  1  1  3  3  3  3  2  2  2  2  2  2  2  1  1  1  1  1  1  3  3  3  3  3  3  3  3  3  3  3  2  2  2  2  2  2  2  2  2  2  2  2  2  2  2  2  2  2  2  2  2  2  2  2  2  2  2  2  2  2  2  2  2  2  2  2  2 |

Aboriginal: N=No, Y=Yes, U=unknown.

Regional: N=No, Y=Yes, U=Unknown.

PorA: Expressed as P1.VR1,VR2.

FHbp MG: FHbp modular group.

FHbp VG: FHbp variant group.

**Table 7. Sequences with no match at the Neisseria Sequence Typing home page.**

>S788fHbp

TGACCCGCCTGCAGCAGCGGAGGGGGTGGTGTCGCCGCCGACATCGGTGCGGGGCTTGCC

GATGCACTAACCGCACCGCTCGACCATAAAGACAAAGGTTTGCAGTCTTTGACGCTGGAT

CAGTCCGTCAGGAAAAACGAGAAACTGAAGCTGGCGGCACAAGGTGCGGAAAAAACTTAT

GGAAACGGTGACAGCCTCAATACGGGCAAATTGAAGAACGACAAGGTCAGCCGTTTCGAC

TTTATCCGCCAAATCGAAGTGGACGGGCAGCTCATTACCTTGGAGAGTGGAGAGTTCCAA

GTATACAAACAAAGCCATTCCGCCTTAACCGCCTTTCAGACCGAGCAAATACAAGATTCG

GAGCATTCCGGGAAGATGGTTGCGAAACGCCAGTTCAGAATCGGCGACATAGCGGGCGAA

CATACATCTTTTGACAAGCTTCCCGAAGGCGGCAGGGCGACATATCGCGGGACGGCGTTC

GGTTCAGACGATGCCGGCGGAAAACTGACCTACACCATAGATTTCGCCGCCAAGCAGGG

>T24fHbp

TTCTGACCGCCTGCAGCAGCGGAGGGGGCGGTGTTGCCGCCGACATCGGTGCGGGGCTTG

CCGATGCACTAACCGCACCGCTCGACCATAAAGACAAAGGTTTGCAGTCTTTGATGCTGG

ATCAGTCCGTCAGGAAAAACGAGAAACTGAAGCTGGCGGCACAAGGTGCGGAAAAAACTT

ATGGAAACGGCGACAGCCTCAATACGGGCAAATTGAAGAACGACAAGGTCAGCCGCTTCG

ACTTTATCCGTCAAATCGAAGTGGACGGGCAGCTCATTACCTTGGAGATCGGAGAGTTCC

AAGTGTACAAACAAAGCCATTCCGCCTTAACCGCCCTTCAGACCGAGCAAGTACAAGACT

CGGAGCATTCGGGAAGATGGTTGCGAAACGCCAGTTCAGAATCGGCGACATAGCGGGCGA

ACATACATCTTTTGACAAGCTTCCCGAAGGCGGCAGGGCGACATATCGCGGGACGGCGTT

CGGTTCAGACGATGCAGGCGGAAAACTGATCTACACCATAGATTTCGCCGCCAAGCAGGG

>T121fHbp

ATTCTGACCGCCTGCAGCAGCGGAGGGGGCGGTGTTGCCGCCGACATCGGTGCGGGGCTT

GCCGATGCACTAACCGCACCGCTCGACCATAAAGACAAAGGTTTGCAGTCTTTGATGCTG

GATCAGTCCGTCAGGAAAAACGAGAAACTGAAGCTGGCGGCACAAGGTGCGGAAAAAACT

TATGGAAACGGCGACAGCCTCAATACGGGCAAATTGAAGAACGACAAGGTCAGCCGCTTC

GACTTTATCCGTCAAATCGAAGTGGACGGGCAGCTCATTACCTTGGAGATCGGAGAGTTC

CAAGTGTACAAACAAAGCCATTCCGCCTTAACCGCCCTTCAGACCGAGCAAGTACAAGAC

TCGGAGCATTCGGGAAGATGGTTGCGAAACGCCAGTTCAGAATCGGCGACATAGCGGGCG

AACATACATCTTTTGACAAGCTTCCCGAAGGCGGCAGGGCGACATATCGCGGGACGGCGT

TCGGTTCAGACGATGCAGGCGGAAAACTGATCTACACCATAGATTTCGCCGCCAAGCA

>J107fetA

TTCCGTAATAATTTCAACGGCGGCAAAACCGTACCGTACAGCGCGCTGGACAAACGCAGC

TACCTCGCCAAAATCGGAACAACCTTCGGCGACGGCGACCACCGCATCGTGTTGAGCCAT

ATGAAAGACCAACACCGAGGCATCCGCACTGTGCGTGAAGAATTTACCGTCGGCAGCGAA

GATTCACGGATAAATATTAAACGCCAAGCCCCTGCTTACCGCGAAACCACACAATCCAAC

ACCAATTTGGCGTACACGGGTAAAAACCTGGGCTTTGTCGAAAAACTGGATGCCAACGCC

TATGTGTTGGAAAAAGAACGCTATTCCGCCGATGACAGCGGCACCGGCTACGCAGGCAAT

GTAAAAGGCCCCAACCATACCCGAATCACCACTCGTGGTGCGAACTTCAACTTCGACAGC

CGCCTTGCCGAACAAACCCTGCTGAAATACGGTATCAACTACCGCCATCAGGAAATCAAA

CCGCAAGCGTTTTTGAATTCACAATTTWAAATTGAAGATAAAGAAAAAGCAACTGATGAA

GAGAAAAATAAGAACCGTGAAAATGAAAAAATTGCCAAAGCCTACCGTCTGACCAACCCG

ACCAAAACCGATGCCGGCGCGTATATCGAAGCCATTCACGAGATTGACGGCTTTACCCTG

ACCGGCGGGCTGCGTTACGACCGCTTCAAGGTGAAAACCCACGACGGCAAAACCGTTTCA

AGCAGCAACCTTAACCCGAGTTTCGGCGTGATTTGGCAGCCGCACGAACACTGGAGCTTC

AGCGCAAGCCACAACTACGCCAGCCGCAGCCCGCGCCTGTATGACGCGCTGCAAACCCAC

GGCAAACGCGGCATCATCTCGATTGCCGACGGCACCAAAGCCGAACGCGCGCGCAATACC

GAAATCGGCTTCAACTAC

>J109fetA

TTCCGTAATAATTTCAACGGCGGCAAAACCGTACCGTACAGCGCGCTGGACAAACGCAGC

TACCTCGCCAAAATCGGAACAACCTTCGGCGACGGCGACCACCGCATCGTGTTGAGCCAT

ATGAAAGACCAACACCGAGGCATCCGCACTGTGCGTGAAGAATTTACCGTCGGCAGCGAA

GATTCACGGATAAATATTAAACGCCAAGCCCCTGCTTACCGCGAAACCACACAATCCAAC

ACCAATTTGGCGTACACGGGTAAAAACCTGGGCTTTGTCGAAAAACTGGATGCCAACGCC

TATGTGTTGGAAAAAGAACGCTATTCCGCCGATGACAGCGGCACCGGCTACGCAGGCAAT

GTAAAAGGCCCCAACCATACCCGAATCACCACTCGTGGTGCGAACTTCAACTTCGACAGC

CGCCTTGCCGAACAAACCCTGCTGAAATACGGTATCAACTACCGCCATCAGGAAATCAAA

CCGCAAGCGTTTTTGAATTCACAATTTAAAWTTGAAGATAAAGAAAAAGCAACTGATGAA

GAGAAAAATAAGAACCGTGAAAATGAAAAAATTGCCAAAGCCTACCGTCTGACCAACCCG

ACCAAAACCGATGCCGGCGCGTATATCGAAGCCATTCACGAGATTGACGGCTTTACCCTG

ACCGGCGGGCTGCGTTACGACCGCTTCAAGGTGAAAACCCACGACGGCAAAACCGTTTCA

AGCAGCAACCTTAACCCGAGTTTCGGCGTGATTTGGCAGCCGCACGAACACTGGAGCTTC

AGCGCAAGCCACAACTACGCCAGCCGCAGCCCGCGCCTGTATGACGCGCTGCAAACCCAC

GGCAAACGCGGCATCATCTCGATTGCCGACGGCACCAAAGCCGAACGCGCGCGCAATACC

GAAATCGGCTTCAACTAC

>J249fetA

TTCCGTAATAATTTCAACGGCGGCAAAACCGTACCGTACAGCGCGCTGGACAAACGCAGC

TACCTCGCCAAAATCGGAACAACCTTCGGCGACGGCGACCACCGCATCGTGTTGAGCCAT

ATGAAAGACCAACACCGAGGCATCCGCACTGTGCGTGAAGAATTTACCGTCGGCAGCGAA

GATTCACGGATAAATATTAAACGCCAAGCCCCTGCTTACCGCGAAACCACACAATCCAAC

ACCAATTTGGCGTACACGGGTAAAAACCTGGGCTTTGTCGAAAAACTGGATGCCAACGCC

TATGTGTTGGAAAAAGAACGCTATTCCGCCGATGACAGCGGCACCGGCTACGCAGGCAAT

GTAAAAGGCCCCAACCATACCCGAATCACCACTCGTGGTGCGAACTTCAACTTCGACAGC

CGCCTTGCCGAACAAATCAACTACCGCCATCAGGAAATCAAACCGCAAGCGTTTTTGAAT

TCACAATTTAAAATTTTGAAGATAAAGAAAAAGCAACTGATGAAGAGAAAAATAAGAACC

GTGAAAATGAAAAAATTGCCAAAGCCTACCGTCTGACCAACCCGACCAAAACCGATGCCG

GCGCGTATATCGAAGCCATTCACGAGATTGACGGCTTTACCCTGACCGGCGGGCTGCGTT

ACGACCGCTTCAAGGTGAAAACCCACGACGGCAAAACCGTTTCAAGCAGCAACCTTAACC

CGAGTTTCGGCGTGATTTGGCAGCCGCACGAACACTGGAGCTTCAGCGCAAGCCACAACT

ACGCCAGCCGCAGCCCGCGCCTGTATGACGCGCTGCAAACCCACGGCAAACGCGGCATCA

TCTCGATTGCCGACGGCACCAAAGCCGAACGCGCGCGCAATACCGAAATCGGCTTCAACT

ACAACGACGGCACGTTTG

>J312fetA

TTCCGTAATAATTTCAACGGCGGCAAAACCGTACCGTACAGCGCGCTGGACAAACGCAGC

TACCTCGCCAAAATCGGAACAAGCTTCGGCGACGGCGACCACCGCATCGTATTGAGCCAT

ATGAAAGACCAGCACCGGGGCATCCGTACCGTCCGTGAAGAATTTACCGTCGGCGGCGAT

AAAGAGCGAATAAGTATGGAACGCCAAGCCCCTGCTTACCGCGAAACCACACAATCCAAC

ACCAATTTGGCGTACACGGGTAAAAACCTGGGCTTTGTCGAAAAACTGGATGCCAACGCC

TATGTGTTGGAAAAAGAACGCTATTCCGCCGATGACAGCGGCACCGGTTACGCAGGCAAT

GTAAAAGGCCCCAACCATACCCAAATCACCACTCGGGGTATGAACTTCAACTTCGACAGC

CGCCTTGCCGAACAAACCCTGCTGAAATACGGTATCAACTACCGCCATCAGGAAATCAAA

CCGCAAGCGTTTTTGAATTCACAATTTAAAATTAATTGAAGATAAAGAAAAAGCAACTGA

TGAAGAGAAAAATAAGAACCGTGAAAATGAAAAAATTGCCAAAGCCTACCGTCTGACCAA

CCCGACCAAAACCGATACCGGCGCGTATATCGAAGCCATTCACGAGATTGACGGCTTTAC

CCTGACCGGCGGGCTGCGTTACGACCGCTTCAAGGTGAAAACCCACGACGGCAAAACCGT

TTCAAGCAACAACCTTAACCCGAGTTTCGGCGTGATTTGGCAGCCGCACGAACACTGGAG

CTTCAGCGCGAGCCACAACTACGCCAGCCGCAGCCCGCGCCTGTATGACGCGCTGCAAAC

CCACGGCAAACGCGGCATCATCTCGATTGCCGACGGCACGAAAGCCGAACGCGCGCGCAA

TACCGAAATCGGCTTCAA

>K104fetA

TTCCGTAATAATTTCAACGGCGGCAAAACCGTACCGTACAGCGCGCTGGACAAACGCAGC

TACCTCGCCAAAATCGGAACAACCTTCGGCGACGGCGACCACCGCATCGTGTTGAGCCAT

ATGAAAGACCAACACCGAGGCATCCGCACTGTGCGTGAAGAATTTACCGTCGGCAGCGAA

GATTCACGGATAAATATTAAACGCCAAGCCCCTGCTTACCGCGAAACCACACAATCCAAC

ACCAATTTGGCGTACACGGGTAAAAACCTGGGCTTTGTCGAAAAACTGGATGCCAACGCC

TATGTGTTGGAAAAAGAACGCTATTCCGCCGATGACAGCGGCACCGGCTACGCAGGCAAT

GTAAAAGGCCCCAACCATACCCGAATCACCACTCGTGGTGCGAACTTCAACTTCGACAGC

CGCCTTGCCGAACAAACCCTGCTGAAATACGGTCAACTACCGCCATCAGGAAATCAAACC

GCAAGCGTTTTTGAATTCACAATTTAAAATTTTGAAGATAAAGAAAAAGCAACTGATGAA

GAGAAAAATAAGAACCGTGAAAATGAAAAAATTGCCAAAGCCTACCGTCTGACCAACCCG

ACCAAAACCGATGCCGGCGCGTATATCGAAGCCATTCACGAGATTGACGGCTTTACCCTG

ACCGGCGGGCTGCGTTACGACCGCTTCAAGGTGAAAACCCACGACGGCAAAACCGTTTCA

AGCAGCAACCTTAACCCGAGTTTCGGCGTGATTTGGCAGCCGCACGAACACTGGAGCTTC

AGCGCAAGCCACAACTACGCCAGCCGCAGCCCGCGCCTGTATGACGCGCTGCAAACCCAC

GGCAAACGCGGCATCATCTCGATTGCCGACGGCACCAAAGCCGAACGCGCGCGCAATACC

GAAATCGGCTTCAACTAC

>K181fetA

TTCCGTAATAATTTCAACGGCGGCAAAACCGTACCGTACAGCGCGCTGGACAAACGCAGC

TACCTCGCCAAAATCGGAACAACCTTCGGCGACGGCGACCACCGCATCGTGTTGAGCCAT

ATGAAAGACCAACACCGAGGCATCCGCACTGTGCGTGAAGAATTTACCGTCGGCAGCGAA

GATTCACGGATAAATATTAAACGCCAAGCCCCTGCTTACCGCGAAACCACACAATCCAAC

ACCAATTTGGCGTACACGGGTAAAAACCTGGGCTTTGTCGAAAAACTGGATGCCAACGCC

TATGTGTTGGAAAAAGAACGCTATTCCGCCGATGACAGCGGCACCGGCTACGCAGGCAAT

GTAAAAGGCCCCAACCATACCCGAATCACCACTCGTGGTGCGAACTTCAACTTCGACAGC

CGCCTTGCCGAACAAACCCTGCTGAAATACGGTATCAACTACCGCCATCAGGAAATCAAA

CCGCAAGCGTTTTTGAATTCACAATTTAAAATTTTGAAGATAAAGAAAAAGCAACTGATG

AAGAGAAAAATAAGAACCGTGAAAATGAAAAAATTGCCAAAGCCTACCGTCTGACCAACC

CGACCAAAACCGATGCCGGCGCGTATATCGAAGCCATTCACGAGATTGACGGCTTTACCC

TGACCGGCGGGCTGCGTTACGACCGCTTCAAGGTGAAAACCCACGACGGCAAAACCGTTT

CAAGCAGCAACCTTAACCCGAGTTTCGGCGTGATTTGGCAGCCGCACGAACACTGGAGCT

TCAGCGCAAGCCACAACTACGCCAGCCGCAGCCCGCGCCTGTATGACGCGCTGCAAACCC

ACGGCAAACGCGGCATCATCTCGATTGCCGACGGCACCAAAGCCGAACGCGCGCGCAATA

CCGAAATCGGCTTCAACT

>K263fetA

TTCCGCAATGTCAACGGCGGCAAAACCGTACCGTACAGCGCGCTGGACAAACGCAGCTAC

CTCGCCAAAATCGGAACAAGCTTCGGCGACGGCGACCACCGCATCGTGTTGAGCCATATG

AAAGACCAACACCGGGGCATCCGCACTGTGCGTGAAGAATTTACCGTCGGCGGCGATAAA

GAGCGAATAAGTATGAAACGCCAAGCCCCATCCTACCGCGAAACCACCCAATCCAACACC

AATTTGGCGTACACCGGCAAAGATTTGGGCTTTGTCGAAAAACTGGATGCCAACGCCTAT

GTGTTGGAAAAAGAACGCTATTCCGCCGATGACAGCGGCACCGGTTACGCAGGCAATGTA

AAAGGCCCCAACCATACCCAAATCACCACTCGGGGTATGAACTTCAACTTCGACAGCCGC

CTTGCCGAACAAACCCTGCTGAAATACGGTATCAACTACCGCCATCAGGAAATCAAACCG

CAAGCGTTTTTGAATTCACAATTTAAAATTTTGAAGATAAAGAAAAAGCAACTGATGAAG

AGAAAAATAAGAACCGTGAAAATGAAAAAATTGCCAAAGCCTACCGTCTGACCAACCCGA

CCAAAACCGATGCCGGCGCGTATATCGAAGCCATTCACGAGATTGACGGCTTTACCCTGA

CCGGCGGGCTGCGTTACGACCGCTTCAAGGTGAAAACCCACGACGGCAAAACCGTTTCAA

GCAGCAACCTTAACCCGAGTTTCGGCGTGATTTGGCAGCCGCACGAACACTGGAGCTTCA

GCGCAAGCCACAACTACGCCAGCCGCAGCCCGCGCCTGTATGACGCGCTGCAAACCCACG

GCAAACGCGGCATCATCTCGATTGCCGACGGCACCAAAGCCGAACGCGCGCGCAATACCG

AAATCGGCTTCAACTACA

>L1fetA

TTCCGTAATAATTTCAACGGCGGCAAAACCGTACCGTACAGCGCGCTGGACAAACGCAGC

TACCTCGCCAAAATCGGAACAAGCTTCGGCGACGGCGACCACCGCATCGTATTGAGCCAT

ATGAAAGACCAGCACCGGGGCATCCGTACCGTCCGTGAAGAATTTACCGTCGGCGGCGAT

AAAGAGCGAATAAGTATGGAACGCCAAGCCCCTGCTTACCGCGAAACCACACAATCCAAC

ACCAATTTGGCGTACACGGGTAAAAACCTGGGCTTTGTCGAAAAACTGGATGCCAACGCC

TATGTGTTGGAAAAAGAACGCTATTCCGCCGATGACAGCGGCACCGGTTACGCAGGCAAT

GTAAAAGGCCCCAACCATACCCAAATCACCACTCGGGGTATGAACTTCAACTTCGACAGC

CGCCTTGCCGAACAAACCCTGCTGAAATACGGTATCAACTACCGCCATCAGGAAATCAAA

CCGCAAGCGTTTTTGAATTCACAATTTAAAATTAATTGAAGATAAAGAAAAAGCAACTGA

TGAAGAGAAAAATAAGAACCGTGAAAATGAAAAAATTGCCAAAGCCTACCGTCTGACCAA

CCCGACCAAAACCGATACCGGCGCGTATATCGAAGCCATTCACGAGATTGACGGCTTTAC

CCTGACCGGCGGGCTGCGTTACGACCGCTTCAAGGTGAAAACCCACGACGGCAAAACCGT

TTCAAGCAACAACCTTAACCCGAGTTTCGGCGTGATTTGGCAGCCGCACGAACACTGGAG

CTTCAGCGCGAGCCACAACTACGCCAGCCGCAGCCCGCGCCTGTATGACGCGCTGCAAAC

CCACGGCAAACGCGGCATCATCTCGATTGCCGACGGCACGAAAGCCGAACGCGCGCGCAA

TACCGAAATCGGCTTCAA

>L265fetA

TTCCGTAATAATTTCAACGGCGGCAAAACCGTACCGTACAGCGCGCTGGACAAACGCAGC

TACCTCGCCAAAATCGGAACAACCTTCGGCGACGGCGACCACCGCATCGTGTTGAGCCAT

ATGAAAGACCAACACCGAGGCATCCGCACTGTGCGTGAAGAATTTACCGTCGGCAGCGAA

GATTCACGGATAAATATTAAACGCCAAGCCCCTGCTTACCGCGAAACCACACAATCCAAC

ACCAATTTGGCGTACACGGGTAAAAACCTGGGCTTTGTCGAAAAACTGGATGCCAACGCC

TATGTGTTGGAAAAAGAACGCTATTCCGCCGATGACAGCGGCACCGGCTACGCAGGCAAT

GTAAAAGGCCCCAACCATACCCGAATCACCACTCGTGGTGCGAACTTCAACTTCGACAGC

CGCCTTGCCGAACAAACCCTGCTGAAATACGGTATCAACTACCGCCATCAGGAAATCAAA

CCGCAAGCGTTTTTGAATTCACAATTTAAAATTAATTGAAGATAAAGAAAAAGCAACTGA

TGAAGAGAAAAATAAGAACCGTGAAAATGAAAAAATTGCCAAAGCCTACCGTCTGACCAA

CCCGACCAAAACCGATGCCGGCGCGTATATCGAAGCCATTCACGAGATTGACGGCTTTAC

CCTGACCGGCGGGCTGCGTTACGACCGCTTCAAGGTGAAAACCCACGACGGCAAAACCGT

TTCAAGCAGCAACCTTAACCCGAGTTTCGGCGTGATTTGGCAGCCGCACGAACACTGGAG

CTTCAGCGCAAGCCACAACTACGCCAGCCGCAGCCCGCGCCTGTATGACGCGCTGCAAAC

CCACGGCAAACGCGGCATCATCTCGATTGCCGACGGCACCAAAGCCGAACGCGCGCGCAA

TACCGAAATCGGCTTCAA

>L552fetA

TTCCGTAATAATTTCAACGGCGGCAAAACCGTACCGTACAGCGCGCTGGACAAACGCAGC

TACCTCGCCAAAATCGGAACAACCTTCGGCGACGGCGACCACCGCATCGTGTTGAGCCAT

ATGAAAGACCAACACCGAGGCATCCGCACTGTGCGTGAAGAATTTACCGTCGGCAGCGAA

GATTCACGGATAAATATTAAACGCCAAGCCCCTGCTTACCGCGAAACCACACAATCCAAC

ACCAATTTGGCGTACACGGGTAAAAACCTGGGCTTTGTCGAAAAACTGGATGCCAACGCC

TATGTGTTGGAAAAAGAACGCTATTCCGCCGATGACAGCGGCACCGGCTACGCAGGCAAT

GTAAAAGGCCCCAACCATACCCGAATCACCACTCGTGGTGCGAACTTCAACTTCGACAGC

CGCCTTGCCGAACAAACCCTGCTGAAATACGGTATCAACTACCGCCATCAGGAAATCAAA

CCGCAAGCGTTTTTGAATTCACAATTTAAAATTAATTGAAGATAAAGAAAAAGCAACTGA

TGAAGAGAAAAATAAGAACCGTGAAAATGAAAAAATTGCCAAAGCCTACCGTCTGACCAA

CCCGACCAAAACCGATGCCGGCGCGTATATCGAAGCCATTCACGAGATTGACGGCTTTAC

CCTGACCGGCGGGCTGCGTTACGACCGCTTCAAGGTGAAAACCCACGACGGCAAAACCGT

TTCAAGCAGCAACCTTAACCCGAGTTTCGGCGTGATTTGGCAGCCGCACGAACACTGGAG

CTTCAGCGCAAGCCACAACTACGCCAGCCGCAGCCCGCGCCTGTATGACGCGCTGCAAAC

CCACGGCAAACGCGGCATCATCTCGATTGCCGACGGCACCAAAGCCGAACGCGCGCGCAA

TACCGAAATCGGCTTCAA

>L658fetA

TTCCGTAATAATTTCAACGGCGGCAAAACCGTACCGTACAGCGCGCTGGACAAACGCAGC

TACCTCGCCAAAATCGGAACAACCTTCGGCGACGGCGACCACCGCATCGTGTTGAGCCAT

ATGAAAGACCAACACCGAGGCATCCGCACTGTGCGTGAAGAATTTACCGTCGGCAGCGAA

GATTCACGGATAAATATTAAACGCCAAGCCCCTGCTTACCGCGAAACCACACAATCCAAC

ACCAATTTGGCGTACACGGGTAAAAACCTGGGCTTTGTCGAAAAACTGGATGCCAACGCC

TATGTGTTGGAAAAAGAACGCTATTCCGCCGATGACAGCGGCACCGGCTACGCAGGCAAT

GTAAAAGGCCCCAACCATACCCGAATCACCACTCGTGGTGCGAACTTCAACTTCGACAGC

CGCCTTGCCGAACAAACCCTGCTGAAATACGGTATCAACTACCGCCATCAGGAAATCAAA

CCGCAAGCGTTTTTGAATTCACAATTTAAAATTAATTGAAGATAAAGAAAAAGCAACTGA

TGAAGAGAAAAATAAGAACCGTGAAAATGAAAAAATTGCCAAAGCCTACCGTCTGACCAA

CCCGACCAAAACCGATGCCGGCGCGTATATCGAAGCCATTCACGAGATTGACGGCTTTAC

CCTGACCGGCGGGCTGCGTTACGACCGCTTCAAGGTGAAAACCCACGACGGCAAAACCGT

TTCAAGCAGCAACCTTAACCCGAGTTTCGGCGTGATTTGGCAGCCGCACGAACACTGGAG

CTTCAGCGCAAGCCACAACTACGCCAGCCGCAGCCCGCGCCTGTATGACGCGCTGCAAAC

CCACGGCAAACGCGGCATCATCTCGATTGCCGACGGCACCAAAGCCGAACGCGCGCGCAA

TACCGAAATCGGCTTCAA

>M510fetA

TTCCGTAATAATTTCAACGGCGGCAAAACCGTACCGTACAGCGCGCTGGACAAACGCAGC

TACCTCGCCAAAATCGGAACAACCTTCGGCGACGGCGACCACCGCATCGTGTTGAGCCAT

ATGAAAGACCAACACCGAGGCATCCGCACTGTGCGTGAAGAATTTACCGTCGGCAGCGAA

GATTCACGGATAAATATTAAACGCCAAGCCCCTGCTTACCGCGAAACCACACAATCCAAC

ACCAATTTGGCGTACACGGGTAAAAACCTGGGCTTTGTCGAAAAACTGGATGCCAACGCC

TATGTGTTGGAAAAAGAACGCTATTCCGCCGATGACAGCGGCACCGGCTACGCAGGCAAT

GTAAAAGGCCCCAACCATACCCGAATCACCACTCGTGGTGCGAACTTCAACTTCGACAGC

CGCCTTGCCGAACAAACCCTGCTGAAATACGGTATCAACTACCGCCATCAGGAAATCAAA

CCGCAAGCGTTTTTGAATTCACAATTTAAAATTAATTGAAGATAAAGAAAAAGCAACTGA

TGAAGAGAAAAATAAGAACCGTGAAAATGAAAAAATTGCCAAAGCCTACCGTCTGACCAA

CCCGACCAAAACCGATGCCGGCGCGTATATCGAAGCCATTCACGAGATTGACGGCTTTAC

CCTGACCGGCGGGCTGCGTTACGACCGCTTCAAGGTGAAAACCCACGACGGCAAAACCGT

TTCAAGCAGCAACCTTAACCCGAGTTTCGGCGTGATTTGGCAGCCGCACGAACACTGGAG

CTTCAGCGCAAGCCACAACTACGCCAGCCGCAGCCCGCGCCTGTATGACGCGCTGCAAAC

CCACGGCAAACGCGGCATCATCTCGATTGCCGACGGCACCAAAGCCGAACGCGCGCGCAA

TACCGAAATCGGCTTCAA

>M526fetA

TTCCGTAATAATTTCAACGGCGGCAAAACCGTACCGTACAGCGCGCTGGACAAACGCAGC

TACCTCGCCAAAATCGGAACAACCTTCGGCGACGGCGACCACCGCATCGTGTTGAGCCAT

ATGAAAGACCAACACCGAGGCATCCGCACTGTGCGTGAAGAATTTACCGTCGGCAGCGAA

GATTCACGGATAAATATTAAACGCCAAGCCCCTGCTTACCGCGAAACCACACAATCCAAC

ACCAATTTGGCGTACACGGGTAAAAACCTGGGCTTTGTCGAAAAACTGGATGCCAACGCC

TATGTGTTGGAAAAAGAACGCTATTCCGCCGATGACAGCGGCACCGGCTACGCAGGCAAT

GTAAAAGGCCCCAACCATACCCGAATCACCACTCGTGGTGCGAACTTCAACTTCGACAGC

CGCCTTGCCGAACAAACCCTGCTGAAATACGGTATCAACTACCGCCATCAGGAAATCAAA

CCGCAAGCGTTTTTGAATTCACAATTTAAAATTAATTGAAGATAAAGAAAAAGCAACTGA

TGAAGAGAAAAATAAGAACCGTGAAAATGAAAAAATTGCCAAAGCCTACCGTCTGACCAA

CCCGACCAAAACCGATGCCGGCGCGTATATCGAAGCCATTCACGAGATTGACGGCTTTAC

CCTGACCGGCGGGCTGCGTTACGACCGCTTCAAGGTGAAAACCCACGACGGCAAAACCGT

TTCAAGCAGCAACCTTAACCCGAGTTTCGGCGTGATTTGGCAGCCGCACGAACACTGGAG

CTTCAGCGCAAGCCACAACTACGCCAGCCGCAGCCCGCGCCTGTATGACGCGCTGCAAAC

CCACGGCAAACGCGGCATCATCTCGATTGCCGACGGCACCAAAGCCGAACGCGCGCGCAA

TACCGAAATCGGCTTCAA

>N648fetA

TTCCGCAATGTCAACGGCGGCAAAACCGTACCGTACAGCGCGCTGGACAAACGCAGCTAC

CTCGCCAAAATCGGAACAAGCTTCGGCGGCGACGACCACCGCATCGTGTTGAGCCATATG

AAAGACCAACACCGGGGCATCCGCACTGTGCGTGAAGAGTTTGCCGTCGGCGACACAAAA

TCACGGATAAATATTAAACGCCAAGCCCCTGCTTACCGCGAAACCACTCAATCCAACACC

AACTTGGCGTACACCGGCAAAGATTTGGGCTTTGTCGAAAAACTGGATGCCAACGCCTAT

GTGTTGGAAAAGAAACGCTATTCCGCCGATGACAAAGATAACGGCTACGCAGGCAATGTA

AAAGGCCCCAACCATACCCGAATCACCACTCGTGGTGCGAACTTCAACTTCGACAGCCGC

CTTGCCGAACAAACCCTGCTGAAATACGGTATCAACTACCGCCATCAGGAAATCAAACCG

CAAGCGTTTTTGAATTCACAATTTAAAATTAATTGAAGATAAAGAAAAAGCAACTGATGA

AGAGAAAAATAAGAACCGTGAAAATGAAAAAATTGCCAAAGCCTACCGTCTGACCAACCC

GACCAAAACCGATGCCGGCGCGTATATCGAAGCCATTCACGAGATTGACGGCTTTACCCT

GACCGGCGGGCTGCGTTACGACCGCTTCAAGGTGAAAACCCACGACGGCAAAACCGTTTC

AAGCAGCAACCTTAACCCGAGTTTCGGCGTGATTTGGCAGCCGCACGAACACTGGAGCTT

CAGCGCAAGCCACAACTACGCCAGCCGCAGCCCGCGCCTGTATGACGCGCTGCAAACCCA

CGGCAAACGCGGCATCATCTCGATTGCCGACGGCACCAAAGCCGAACGCGCGCGCAATAC

CGAAATCGGCTTCAACTA

>N321fetA

TTCCGTAATAATTTCAACGGCGGCAAAACCGTACCGTACAGCGCGCTGGACAAACGCAGC

TACCTCGCCAAAATCGGAACAACCTTCGGCGACGGCGACCACCGCATCGTGTTGAGCCAT

ATGAAAGACCAACACCGAGGCATCCGCACTGTGCGTGAAGAATTTACCGTCGGCAGCGAA

GATTCACGGATAAATATTAAACGCCAAGCCCCTGCTTACCGCGAAACCACACAATCCAAC

ACCAATTTGGCGTACACGGGTAAAAACCTGGGCTTTGTCGAAAAACTGGATGCCAACGCC

TATGTGTTGGAAAAAGAACGCTATTCCGCCGATGACAGCGGCACCGGCTACGCAGGCAAT

GTAAAAGGCCCCAACCATACCCGAATCACCACTCGTGGTGCGAACTTCAACTTCGACAGC

CGCCTTGCCGAACAAACCCTGCTGAAATACGGTATCAACTACCGCCATCAGGAAATCAAA

CCGCAAGCGTTTTTGAATTCACAATTTAAAATTAATTGAAGATAAAGAAAAAGCAACTGA

TGAAGAGAAAAATAAGAACCGTGAAAATGAAAAAATTGCCAAAGCCTACCGTCTGACCAA

CCCGACCAAAACCGATGCCGGCGCGTATATCGAAGCCATTCACGAGATTGACGGCTTTAC

CCTGACCGGCGGGCTGCGTTACGACCGCTTCAAGGTGAAAACCCACGACGGCAAAACCGT

TTCAAGCAGCAACCTTAACCCGAGTTTCGGCGTGATTTGGCAGCCGCACGAACACTGGAG

CTTCAGCGCAAGCCACAACTACGCCAGCCGCAGCCCGCGCCTGTATGACGCGCTGCAAAC

CCACGGCAAACGCGGCATCATCTCGATTGCCGACGGCACCAAAGCCGAACGCGCGCGCAA

TACCGAAATCGGCTTCAA

>P297fetA

TTCCGTAATAATTTCAACGGCGGCAAAACCGTACCGTACAGCGCGCTGGACAAACGCAGC

TACCTCGCCAAAATCGGAACAACCTTCGGCGACGGCGACCACCGCATCGTGTTGAGCCAT

ATGAAAGACCAACACCGAGGCATCCGCACTGTGCGTGAAGAATTTACCGTCGGCAGCGAA

GATTCACGGATAAATATTAAACGCCAAGCCCCTGCTTACCGCGAAACCACACAATCCAAC

ACCAATTTGGCGTACACGGGTAAAAACCTGGGCTTTGTCGAAAAACTGGATGCCAACGCC

TATGTGTTGGAAAAAGAACGCTATTCCGCCGATGACAGCGGCACCGGCTACGCAGGCAAT

GTAAAAGGCCCCAACCATACCCGAATCACCACTCGTGGTGCGAACTTCAACTTCGACAGC

CGCCTTGCCGAACAAACCCTGCTGAAATACGGTATCAACTACCGCCATCAGGAAATCAAA

CCGCAAGCGTTTTTGAATTCACAATTTAAAATTAATTGAAGATAAAGAAAAAGCAACTGA

TGAAGAGAAAAATAAGAACCGTGAAAATGAAAAAATTGCCAAAGCCTACCGTCTGACCAA

CCCGACCAAAACCGATGCCGGCGCGTATATCGAAGCCATTCACGAGATTGACGGCTTTAC

CCTGACCGGCGGGCTGCGTTACGACCGCTTCAAGGTGAAAACCCACGACGGCAAAACCGT

TTCAAGCAGCAACCTTAACCCGAGTTTCGGCGTGATTTGGCAGCCGCACGAACACTGGAG

CTTCAGCGCAAGCCACAACTACGCCAGCCGCAGCCCGCGCCTGTATGACGCGCTGCAAAC

CCACGGCAAACGCGGCATCATCTCGATTGCCGACGGCACCAAAGCCGAACGCGCGCGCAA

TACCGAAATCGGCTTCAA

>Q203fetA

TTCCGTAATAATTTCAACGGCGGCAAAACCGTACCGTACAGCGCGCTGGACAAACGCAGC

TACCTCGCCAAAATCGGAACAACCTTCGGCGACGGCGACCACCGCATCGTGTTGAGCCAT

ATGAAAGACCAACACCGAGGCATCCGCACTGTGCGTGAAGAATTTACCGTCGGCAGCGAA

GATTCACGGATAAATATTAAACGCCAAGCCCCTGCTTACCGCGAAACCACACAATCCAAC

ACCAATTTGGCGTACACGGGTAAAAACCTGGGCTTTGTCGAAAAACTGGATGCCAACGCC

TATGTGTTGGAAAAAGAACGCTATTCCGCCGATGACAGCGGCACCGGCTACGCAGGCAAT

GTAAAAGGCCCCAACCATACCCGAATCACCACTCGTGGTGCGAACTTCAACTTCGACAGC

CGCCTTGCCGAACAAACCCTGCTGAAATACGGTATCAACTACCGCCATCAGGAAATCAAA

CCGCAAGCGTTTTTGAATTCACAATTTAAAATTTTGAAGATAAAGAAAAAGCAACTGATG

AAGAGAAAAATAAGAACCGTGAAAATGAAAAAATTGCCAAAGCCTACCGTCTGACCAACC

CGACCAAAACCGATGCCGGCGCGTATATCGAAGCCATTCACGAGATTGACGGCTTTACCC

TGACCGGCGGGCTGCGTTACGACCGCTTCAAGGTGAAAACCCACGACGGCAAAACCGTTT

CAAGCAGCAACCTTAACCCGAGTTTCGGCGTGATTTGGCAGCCGCACGAACACTGGAGCT

TCAGCGCAAGCCACAACTACGCCAGCCGCAGCCCGCGCCTGTATGACGCGCTGCAAACCC

ACGGCAAACGCGGCATCATCTCGATTGCCGACGGCACCAAAGCCGAACGCGCGCGCAATA

CCGAAATCGGCTTCAACT

>S697fetA

TTCCGTAATAATTTCAACGGCGGCAAAACCGTACCGTACAGCGCGCTGGACAAACGCAGC

TACCTCGCCAAAATCGGAACAACCTTCGGCGACGGCGACCACCGCATCGTGTTGAGCCAT

ATGAAAGACCAACACCGAGGCATCCGCACTGTGCGTGAAGAATTTACCGTCGGCAGCGAA

GATTCACGGATAAATATTAAACGCCAAGCCCCTGCTTACCGCGAAACCACACAATCCAAC

ACCAATTTGGCGTACACGGGTAAAAACCTGGGCTTTGTCGAAAAACTGGATGCCAACGCC

TATGTGTTGGAAAAAGAACGCTATTCCGCCGATGACAGCGGCACCGGCTACGCAGGCAAT

GTAAAAGGCCCCAACCATACCCGAATCACCACTCGTGGTGCGAACTTCAACTTCGACAGC

CGCCTTGCCGAACAAACCCTGCTGAAATACGGTATCAACTACCGCCATCAGGAAATCAAA

CCGCAAGCGTTTTTGAATTCACAATTTAAAATTATTGAAGATAAAGAAAAAGCAACTGAT

GAAGAGAAAAATAAGAACCGTGAAAATGAAAAAATTGCCAAAGCCTACCGTCTGACCAAC

CCGACCAAAACCGATGCCGGCGCGTATATCGAAGCCATTCACGAGATTGACGGCTTTACC

CTGACCGGCGGGCTGCGTTACGACCGCTTCAAGGTGAAAACCCACGACGGCAAAACCGTT

TCAAGCAGCAACCTTAACCCGAGTTTCGGCGTGATTTGGCAGCCGCACGAACACTGGAGC

TTCAGCGCAAGCCACAACTACGCCAGCCGCAGCCCGCGCCTGTATGACGCGCTGCAAACC

CACGGCAAACGCGGCATCATCTCGATTGCCGACGGCACCAAAGCCGAACGCGCGCGCAAT

ACCGAAATCGGCTTCAAC

>T233fetA

TTCCGTAATAATTTCAACGGCGGCAAAACCGTACCGTACAGCGCGCTGGACAAACGCAGC

TACCTCGCCAAAATCGGAACAACCTTCGGCGACGGCGACCACCGCATCGTGTTGAGCCAT

ATGAAAGACCAACACCGAGGCATCCGCACTGTGCGTGAAGAATTTACCGTCGGCAGCGAA

GATTCACGGATAAATATTAAACGCCAAGCCCCTGCTTACCGCGAAACCACACAATCCAAC

ACCAATTTGGCGTACACGGGTAAAAACCTGGGCTTTGTCGAAAAACTGGATGCCAACGCC

TATGTGTTGGAAAAAGAACGCTATTCCGCCGATGACAGCGGCACCGGCTACGCAGGCAAT

GTAAAAGGCCCCAACCATACCCGAATCACCACTCGTGGTGCGAACTTCAACTTCGACAGC

CGCCTTGCCGAACAAACCCTGCTGAAATACGGTATCAACTACCGCCATCAGGAAATCAAA

CCGCAAGCGTTTTTGAATTCACAATTTAAAATTTGAAGATAAAGAAAAAGCAACTGATGA

AGAGAAAAATAAGAACCGTGAAAATGAAAAAATTGCCAAAGCCTACCGTCTGACCAACCC

GACCAAAACCGATGCCGGCGCGTATATCGAAGCCATTCACGAGATTGACGGCTTTACCCT

GACCGGCGGGCTGCGTTACGACCGCTTCAAGGTGAAAACCCACGACGGCAAAACCGTTTC

AAGCAGCAACCTTAACCCGAGTTTCGGCGTGATTTGGCAGCCGCACGAACACTGGAGCTT

CAGCGCAAGCCACAACTACGCCAGCCGCAGCCCGCGCCTGTATGACGCGCTGCAAACCCA

CGGCAAACGCGGCATCATCTCGATTGCCGACGGCACCAAAGCCGAACGCGCGCGCAATAC

CGAAATCGGCTYCAACTA

>P450fetA

TTCCGCAATGACAACGGCGGCAAAACCGTACCGTACAGCGCGCTGGACAAACGCAGCTAC

CTCGCCAAAATCGGAACAAGCTTCGGCGACGACGACCACCGCATCGTATTGAGCCACATG

AAAGACCAACACCGAGGCATCCGCACTGTGCGTGAAGAGTTTGCCGTCAGCGAAAAAAAT

TCACGGATAACTATTAAACGCCAAGCCCCTGCTTACCGCGAAACCACTCAATCCAACACC

AACTTGGCGTACACCGGCAAAGATTTGGGCTTTGTCGAAAAACTGGATGCCAACGCCTAT

GTGTTGGAAAAGAAACGCTATTCCGCCGATGACAAAGATAACGGCTACGCAGGCAATGTA

AAAGGCCCTAACCATACCCGAATCACCACTCGAGGTATGAATTTCAACTTCGACAGCCGC

CTTGCCGAACAAACCCTGTTGAAATACGSTWTCAACTACCGCCATCAGGAAATCAAACCG

CAAGCGTTTTTGAATTCACAATTTWAAATTGAAGATAAAAAAGATGCAACTGATGAAGAG

AAAAAGAAGAACCGTGACAATGAAAACATTGCCAAAGCCTACCGTCTGACCAACCCGACC

AAAACCGATACCGGCGCGTATATCGAAGCCATTCACGAAATTAACGGCTTTACCCTGACC

GGCGGGCTGCGTTACGACCGCTTCAAGGTGAAAACCCATGACGGCAAAACCGTTTCAAGC

AGCAGCCTTAACCCGAGTTTCGGTGTAATTTGGCAGCCGCACGAATACTGGAGCTTCAGC

GCGAGCCACAACTACGCCAGCCGCAGCCCGCGCCTGTATGACGCGCTGCAAACCCACGGC

AAGCGCGGCATCATCTCGATTGCTGACGGCACCAAAGCCGAACGCGCGCGCAATACCGAA

ATCGGCTTCAACTACAAC

>P583fetA

TTCCGCAATGACAACGGCGGCAAAACCGTACCGTACAGCGCGCTGGACAAACGCAGCTAC

CTCGCCAAAATCGGAACAAGCTTCGGCGACGACGACCACCGCATCGTATTGAGCCACATG

AAAGACCAACACCGAGGCATCCGCACTGTGCGTGAAGAGTTTGCCGTCAGCGAAAAAAAT

TCACGGATAACTATTAAACGCCAAGCCCCTGCTTACCGCGAAACCACTCAATCCAACACC

AACTTGGCGTACACCGGCAAAGATTTGGGCTTTGTCGAAAAACTGGATGCCAACGCCTAT

GTGTTGGAAAAGAAACGCTATTCCGCCGATGACAAAGATAACGGCTACGCAGGCAATGTA

AAAGGCCCTAACCATACCCGAATCACCACTCGAGGTATGAATTTCAACTTCGACAGCCGC

CTTGCCGAACAAACCCTGTTGAAATACGGTATCAACTACCGCCATCAGGAAATCAAACCG

CAAGCGTTTTTGAATTCACAATTTAAAATTATTTAAATTGAAGATAAAAAAGATGCAACT

GATGAAGAGAAAAAGAAGAACCGTGACAATGAAAACATTGCCAAAGCCTACCGTCTGACC

AATCCGACCAAAACCGATACCGGCGCGTATATCGAAGCCATTCACGAGATTGACGGCTTT

ACCCTGACCGGCGGGCTGCGTTACGACCGCTTCAAGGTGAAAACCCACGACGGCAAAACC

GTTTCAAGCAGCAACCTTAACCCGAGTTTCGGCGTGATTTGGCAGCCGCACGAACACTGG

AGCTTCAGCGCGAGCCACAACTACGCCAGCCGCAGCCCGCGCCTGTATGACGCGCTGCAA

ACCCACGGCAAGCGCGGCATCATTTCGATTGCCGACGGCACGAAAGCCGAACGCGCGCGC

AATACCGAAATCGGCTTC

>S504fetA

TTCCGTAATTTCAACGGAGGCAAAACCGTACCTTACAGCGCACTGGACAAACGCAGCTAC

CTCGCCAAAATCGGAACAACCTTCGGCGACGGCGACCACCGCATCGTATTGAGCCACATG

AAAGACCAGCACCGAGGCATCCGTACCGTCCGTGAAGAATTTACCGTTGGCGACGCAAGT

TCACGGACAAATATTACACGCCAAGCCCCATCCTACCGCGAAACCACACAATCCAACACC

AACTTGGAGTACACGGGTAAAAACCTGGGCTTTGTCGAAAAACTGAATGCCAACGCCTAT

GTGTTGGAAAACGAGCGCTATTCCGCCGATGACAGCGGCAGCGGTTACGCAGGCAATGTG

GTCGGCCCTAACCACACCCGAATCGCCACTCGTGGTGCGAACTTCAACTTCGACAGCCGC

CTTGCCGAACAAACCCTGTTGAAATACGGTATCAACTACCGCCATCAGGAAATCAAACCR

CAAGCATTTTTGAATTCACAATTTAAAATTGAAGATAAAAAAGATGCAACTGAGGAAGAT

AAAAATAAGAACCGTGCAAATGAAGCCTTTGTCAAAGCTTACCACCTGACTAATCCGACC

AAAACGGATGCCGGCGCATATATCGAAGCCATTCACGAGATTGACGGCTTTACCCTGACC

GGCGGGTTGCGTTACGACCGCTTCAAGGTTAAAACCCATGACGGCAAAACCGTTTCAAGC

AGCAGCCTTAACCCGAGTTTCGGTGTGATTTGGCAGCCGCACGAATACTGGAGCTTCAGC

GCAAGCCACAACTACGCCAGCCGCAGCCCGCGCCTGTACGACGCGCTGCAAACCCACGGC

AAACGCGGCATCATCTCGATTGCCGACGGTACGAAAGCCGAACGCGCGCGCAATACCGAA

ATCGGCTTCAACTACAAC

>P247fetA

TTCCGCAATGACAACGGCGGCAAAACCGTACCGTACAGCGCGCTGGACAAACGCAGCTAC

CTCGCCAAAATCGGAACAAGCTTCGGCGACGACGACCACCGCATCGTATTGAGCCACATG

AAAGACCAACACCGAGGCATCCGCACTGTGCGTGAAGAGTTTGCCGTCAGCGAAAAAAAT

TCACGGATAACTATTAAACGCCAAGCCCCTGCTTACCGCGAAACCACTCAATCCAACACC

AACTTGGCGTACACCGGCAAAGATTTGGGCTTTGTCGAAAAACTGGATGCCAACGCCTAT

GTGTTGGAAAAGAAACGCTATTCCGCCGATGACAAAGATAACGGCTACGCAGGCAATGTA

AAAGGCCCTAACCATACCCGAATCACCACTCGAGGTATGAATTTCAACTTCGACAGCCGC

CTTGCCGAACAAACCCTGTTGAAATACGGTATCAACTACCGCCATCAGGAAATCAAACCG

CAAGCGTTTTTGAATTCGAATTTAAAATTGAAGATAAAACTGGTGCAACCCCGGCAGAAA

TACAAAAAAACCGTGCAGATGAAGCCACTGTTCACGCCTACAAACTTTCCAACCCAACCA

AAACCGATACCGGCGCGTATATCGAAGCCATTCACGAAATTAACGGCTTTACCCTGACCG

GCGGGCTGCGTTACGACCGCTTCAAGGTGAAAACCCACGACGGCAAAACCGTTTCAAGCA

GCAACCTTAACCCGAGTTTCGGCGTGATTTGGCAGCCGCACGAACACTGGAGCTTCAGCG

CGAGCCACAACTACGCCAGCCGCAGCCCGCGCCTGTATGACGCGCTGCAAACCCACGGCA

AACGCGGCATCATCTCGATTGCCGACGGCACCAAAGCCGAACGCGCGCGCAATACCGAAA

TCGGCTTCAACTACAACG

>P459fetA

TTCCGCAATGACAACGGCGGCAAAACCGTACCGTACAGCGCGCTGGACAAACGCAGCTAC

CTCGCCAAAATCGGAACAAGCTTCGGCGACGACGACCACCGCATCGTATTGAGCCACATG

AAAGACCAACACCGAGGCATCCGCACTGTGCGTGAAGAGTTTGCCGTCAGCGAAAAAAAT

TCACGGATAACTATTAAACGCCAAGCCCCTGCTTACCGCGAAACCACTCAATCCAACACC

AACTTGGCGTACACCGGCAAAGATTTGGGCTTTGTCGAAAAACTGGATGCCAACGCCTAT

GTGTTGGAAAAGAAACGCTATTCCGCCGATGACAAAGATAACGGCTACGCAGGCAATGTA

AAAGGCCCTAACCATACCCGAATCACCACTCGAGGTATGAATTTCAACTTCGACAGCCGC

CTTGCCGAACAAACCCTGTTGAAATACGSTWTCAACTACCGCCATCAGGAAATCAAACCG

CAAGCGTTTTTGAATTCACAATTTAAAATTTTTAAATTGAAGATAAAAAAGATGCAACTG

ATGAAGAGAAAAAGAAGAACCGTGACAATGAAAACATTGCCAAAGCCTACCGTCTGACCA

ACCCGACCAAAACCGATACCGGCGCGTATATCGAAGCCATTCACGAGATTGACGGCTTTA

CCCTGACCGGCGGGCTGCGTTACGACCGCTTCAAGGTGAAAACCCACGACGGCAAAACCG

TTTCAAGCAGCAACCTTAACCCGAGTTTCGGCGTGATTTGGCAGCCGCACGAACACTGGA

GCTTCAGCGCGAGCCACAACTACGCCAGCCGCAGCCCGCGCCTGTATGACGCGCTGCAAA

CCCACGGCAAGCGCGGCATCATTTCGATTGCCGACGGCACGAAAGCCGAACGCGCGCGCA

ATACCGAAATCGGCTTCA

>Q390fetA

TTCCGCAATGTCAACGGCGGCAAAACCGTACCGTACAGCGCACTGGACAAACGCAGCTAC

CTCGCCAAAATCGGAACAAGCTTCGGCGGCGACGACCACCGCATCGTGTTGAGCCATATG

AAAGACCAACACCGAGGCATCCGCACTGTGCGTGAAGAATTTACCGTCGGCGGCGACGAT

TCACGGATAAATATTAAACGCCAAGCCCCTGCTTACCGCGAAACCACCCAATCCAACACC

AACTTGGCGTACACCGGCAAAGATTTGGGCTTTGTCGAAAAACTGGATGCCAACGCCTAT

GTGTTGGAAAAAGAACGCTATTCCGCCGATGACAGCGGCACCGGCTACGCAGGCAATGTA

AAAGGCCCCAACCATACCCAAATCACCACTCGTGGTGCGAACTTCAACTTCGACAGCCGC

CTTGCCGAACAAATCAACTACCGCCATCAGGAAATCAAACCGCAAGCGTTTTTGAATTCA

CAATTTAAAATTAATTGAAGATAAAAAAGATGCAACTGATGAAGAGAAAAAGAAGAACCG

TGACAATGAAAACATTGCCAAAGCCTACCGTCTGACCAACCCGACCAAAACCGATACCGG

CGCGTATATCGAAGCCATTCACGAGATTGACGGCTTTACCCTGACCGGCGGGCTGCGTTA

CGACCGCTTCAAGGTGAAAACCCACGACGGCAAAACCGTTTCAAGCAGCAACCTTAACCC

GAGTTTCGGTGTGATTTGGCAGCCGCACGAACACTGGAGCTTCAGCGCGAGCCACAACTA

CGCCAGCCGCAGCCCGCGCCTGTATGACGCGCTGCAAACCCACGGTAAACGCGGCATCAT

CTCGATTGCCGACGGCACGAAAGCCGAACGCGCACGCAATACCGAAATCGGCTTCAACTA

CAACGACGGCACGTTTGC

>T416fetA

TTCCGTAATAATTTCAACGGCGACAAGACCGTACCGTACAGCGCGCTGGACAAACGCAGC

TACCTCGCCAAAATCGGAACAACCTTCGGCGACGGCGACCACCGCATCGTGTTGAGCCAC

ATGAAAGACCAACACCGAGGCATCCGTACCGTCCGTGAAGAATTTACCGTCGGCGACGAA

GATTCACGGATAAATATTGGCCGCCAAGCCCCTGCTTACCGCGAAACCACCCAATCCAAC

ACCAATTTGGAGTACACGGGTAAAAACCTGGGCTTTGTCGAAAAACTGGATGCCAACGCC

TATGTGTTGGAAAAAGAACGCTATTCCGCCGATGACAGCGGCACCGGCTACGCAGGCAAT

GTAAAAGGCCCCAACCATACCCAAATCACCACTCGGGGTATGAACTTCAACTTCGACAGC

CGCCTTGCCGAACAAACCCTGCTGAAATACGGTATCAACTACCGCCATCAGGAAATCAAA

CCGCAAGCGTTTTTGAACTCGAAATTCTCCATCCCAGACGACAGAAAAGAAAAACGGTCA

AGATGTTGCTAAACCAGCAGACCAACAAGCCAAAGACCGCAAAGACGAAGCCCTTGTCCA

TTCCTACCGTCTGACCAACCCGACCAAAACCGATACCGGCGCGTATATCGAAGCCATTCA

CGAAATTAACGGCTTTACCCTGACCGGCGGGCTGCGTTACGACCGCTTCAAGGTGAAAAC

CCACGACGGCAAAACCGTTTCAAGCAGCAACCTTAACCCGAGTTTCGGCGTGATTTGGCA

GCCGCACGAACACTGGAGCTTCAGCGCGAGCCACAACTACGCCAGCCGTAGCCCGCGCCT

GTATGACGCGCTGCAAACCCACGGCAAACGCGGCATCATCTCGATTGCCGACGGCACGAA

AGCCGAACGCGCGCGCAA

>L223fetA

TTCCGCAATGTCAACGGCGGCAAAACCGTACCGTACAGCGCGCTGGACAAACGCAGCTAC

CTCGCCAAAATCGGAACAACCTTCGGCGACGGCGACCACCGCATCGTGTTGAGCCATATG

AAAGACCAACACCGGGGCATCCGCACTGTGCGTGAAGAATTTACCGTCGGCAGCGAAGAT

TCACGGATAAATATTAAACGCCAAGCCCCTGCTTACCGCGAAACCACACAATCCAACACC

AATTTGGCGTACACGGGTAAAAACCTGGGCTTTGTCGAAAAACTGGATGCCAACGCCTAT

GTGTTGGAAAAAGAACGCTATTCCGCCGATGACAGCGGCACCGGCTACGCAGGCAATGTA

AAAGGCCCCAACCATACCCGAATCACCACTCGTGGTGCGAACTTCAACTTCGACAGCCGC

CTTGCCGAACAAACCCTGCTGAAATACGGTATCAACTACCGCCATCAGGAAATCAAACCG

CATTTAAGATCTCCGATAAGAAACCAGACCCAAACAATCCCGGAAAAGAAATAGCTAAGA

CCGATGAAGAAAAAGCGAAAGACAAGATAGATATGGCTCTTGTCCATTCCTACAAACTGT

CTAACCCAGCCAAAACCGATACCGGCGCGTATATCGAAGCCATTCACGAAATTAACGGCT

TTACCCTGACCGGCGGGCTGCGTTACGACCGCTTCAAGGTGAAAACCCATGACGGCAAAA

CTGTTTCAAGCAGCAACCTTAACCCGAGTTTCGGTGTGATTTGGCAGCCGCACGAACACT

GGAGCTTCAGCGCGAGCMACAACTACGCCAGCCGCAGCCCGCGCCTGTATGACGCGCTGC

AAACCCACGGTAAACGCGGCATCATCTCGATTGCCGACGSMMSGAAAGCCGAACGCGCGC

GCAATACCGAAATCGGCT

>W233fetA

TTCCGTAATAATTTCAACGGCGGCAAAACCGTACCGTACAGCGCACTGGACAAACGCAGC

TACCTCGCCAAAATCGGAACAACCTTCGGCGACGGCGACCACCGCATCGTATTGAGCCAT

ATGAAAGACCAGCACCGGGGCATCCGTACCGTCCGTGAAGAATTTACCGTCGGCGGCGAT

AAAGAGCGAATAAGTATGAAACGCCAAGCCCCATCCTACCGCGAAACCACCCAATCCAAC

ACCAACTTGGCGTACACCGGCAAAGATTTGGGCTTTGTCGAAAAACTGGATGCCAATGTT

TATGTGTTGGAAAAGAAACGCTATTCCGCCGATGACAGCGGCACCGGCTACGCAGGCAAT

GTAAAAGGCCCTAACCATACCCGAATCACTACTCGGGGTATGAATTTCAACTTCGACAGC

CGCCTTGCCGAACAAACCCTGTTGATTTAAGATCTCCGATAAGAAACCAGACCCAAACAA

TCCCGGAAAGAAATAGCTAAGACCGATGAAGAAAAAGCGAAAGACAAGATAGATATGGCT

CTTGTCCATTCCTACAAACTGTCTAACCCAGCCAAAACCGATACCGGCGCGTATATCGAA

GCCATTCACGAAATTAACGGCTTTACCCTGACCGGCGGGCTGCGTTACGACCGCTTCAAG

GTGAAAACCCATGACGGCAAAACTGTTTCAAGCAGCAACCTTAACCCGAGTTTCGGTGTG

ATTTGGCAGCCGCACGAACACTGGAGCTTCAGCGCGAGCCACAACTACGCCAGCCGCAGC

CCGCGCCTGTATGACGCGCTGCAAACCCACGGTAAACGCGGCATCATCTCGATTGCCGAC

GGCACGAAAGCCGAACGCGCGCGCAATACCGAAATCGGCTTCAACTACAACGACGGCA

>L617fetA

TTCCGCAATGTCAACGGCGGCAAAACCGTACCGTACAGCGCGCTGGACAAACGCAGCTAC

CTCGCCAAAATCGGAACAACCTTCGGCGACGACGACCACCGCATCGTGTTGAGCCATATG

AAAGACCAACACCGAGGCATCCGCACTGTGCGTGAAGAATTTACCGTCGGCGACACAAGT

TCACGGATAAATATTACACGCCAAGCCCCTGCTTACCGCGAAACTACCCAATCCAACACC

AACTTGGCGTACACGGGTAAAAACCTGGGCTTTGTCGAAAAACTGGATGCCAACGCCTAT

GTGTTGGAAAAAGAACGCTATTCCGCCGATGACAGCGGCACCGGCTACGCAGGCAATGTA

AAAGGCCCCAACCATACCCGAATCACCACTCGTGGTGCGAACTTCAACTTCGACAGCCGC

CTTGCCGAACAAACCCTGTTGAAATACGGTATCAACTACCGCCATCAGGAAATCAAACCG

CAAGCGTTTTTGAACGGCGAATTTGAGATCTCCGGTAAGAAGAAAGATCCGAAAGATCCC

AAAAAAGAAATAGATAAGACCGCTGAAGAAAAAGAAAAAGACAAGAAAGATATGGCTCTT

GTCCACTCCTACAAACTTTCCAACCCAGCCAAAACGGATACCGGCGCGTATATCGAAGCC

ATTCACGACATCGGCGATTTCACGCTGACCGGCGGGCTGCGTTACGACCGCTTCAAGGTG

AAAACCCACGACGGCAAAACCGTTTCAAGCAACAACCTTAACCCGAGTTTCGGCGTGATT

TGGCAGCCGCACGAACACTGGAGCTTCAGCGCGAGCCACAACTACGCCAGCCGCAGCCCG

CGCCTGTATGACGCGCTGCAAACCCACGGCAAACGCGGCATCATCTCGATTGCCGACGGC

ACGAAAGCCGAACGCGCG

>P396fetA

TTCCGCAATGTCAACGGCGGCAAAACCGTACCGTACAGCGCGCTGGACAAACGCAGCTAC

CTCGCCAAAATCGGAACAACCTTCGGCGACGACGACCACCGCATCGTGTTGAGCCATATG

AAAGACCAACACCGAGGCATCCGCACTGTGCGTGAAGAATTTACCGTCGGCGACACAAGT

TCACGGATAAATATTACACGCCAAGCCCCTGCTTACCGCGAAACTACCCAATCCAACACC

AACTTGGCGTACACGGGTAAAAACCTGGGCTTTGTCGAAAAACTGGATGCCAACGCCTAT

GTGTTGGAAAAAGAACGCTATTCCGCCGATGACAGCGGCACCGGCTACGCAGGCAATGTA

AAAGGCCCCAACCATACCCGAATCACCACTCGTGGTGCGAACTTCAACTTCGACAGCCGC

CTTGCCGAACAAACCCTGTTGAAATACGGTATCAACTACCGCCATCAGGAAATCAAACCG

CAAGCGTTTTTGAACGGCGAATTTGAGATCTCCGGTAAGAAGAAAGATCCGAAAGATCCC

AAAAAAGAAATAGATAAGACCGCTGAAGAAAAAGAAAAAGACAAGAAAGATATGGCTCTT

GTCCACTCCTACAAACTTTCCAACCCAGCCAAAACGGATACCGGCGCGTATATCGAAGCC

ATTCACGACATCGGCGATTTCACGCTGACCGGCGGGCTGCGTTACGACCGCTTCAAGGTG

AAAACCCACGACGGCAAAACCGTTTCAAGCAACAACCTTAACCCGAGTTTCGGCGTGATT

TGGCAGCCGCACGAACACTGGAGCTTCAGCGCGAGCCACAACTACGCCAGCCGCAGCCCG

CGCCTGTATGACGCGCTGCAAACCCACGGCAAACGCGGCATCATCTCGATTGCCGACGGC

ACGAAAGCCGAACGCGCG

>K274fetA

TTCCGTAATTTCAACGGCGGCAAAACCGTACCGTACAGCGCACTGGACAAACGCAGCTAC

CTGGCCAAAATCGGAACAACCTTCGGCGACGGCGACCACCGCATCGTATTGAGCCACATG

AAAGACCAGCACCGAGGCATCCGTACCGTCCGTGAAGAATTTACCGTTGGCGACGCAAGT

TCACGGACAAATATTACACGCCAAGCCCCATCCTACCGCGAAACCACACAATCCAACACC

AACTTGGCGTACACCGGCAAAGATTTGGGCTTTGTCGAAAAACTGGATGCCAACGCCTAT

GTGTTGGAAAAGAAACGCTATTCCGCCGATGACAGCGGCAGCGGTTACGCAGGCAATGTG

GTCGGCCCTAACCATACTCGAATCGCCACTCGTGGTGCGAACTTCAACTTCGACAGCCGC

CTTGCCGAACAAATCAAYTACCGCCATCAGGAAATCAAACCGCAAGCGTTTTTGAATTCG

CAATTTAAAATTGAAGATAAAACTGGTGCAACCCCGGCAGAAATACAAAAAAACCGTGCA

GATGAAGCCACTGTTCACGCCTACAAACTTTCCAACCCAACCAAAACCGATACCGGCGCG

TATATCGAAGCCATTCACGAAATTAACGGCTTTACCCTGACCGGCGGGCTGCGTTACGAC

CGCTTCAAGGTGAAAACCCACGACGGCAAAACCGTTTCAAGCAGCAACCTTAACCCGAGT

TTCGGCGTGATTTGGCAGCCGCACGAACACTGGAGCTTCAGCGCGAGCCACAACTACGCC

AGCCGCAGCCCGCGCCTGTATGACGCGCTGCAAACCCACGGCAAACGCGGCATCATCTCG

ATTGCCGACGGCACCAAAGCCGAACGCGCGCGCAATACCGAAATCGGCTTCAACTACAAC

G

>N461fetA

TCAACTACCGCCATCAGGAAATCAAACCGCAAGCGTTTTTGAATTCACAATTTAAAATTA

ATTGAAGATAAAGAAAAAGCAACTGATGAAGAGAAAAATAAGAACCGTGAAAATGAAAAA

ATTGCCAAAGCCTACCGTCTGACCAACCCGACCAAAACCGATGCCGGCGCGTATATCGAA

GCCATTCACGAGATTGACGGCTTTACCCTGACCGGCGGGCTGCGTTACGACCGCTTCAAG

GTGAAAACCCACGACGGCAAAACCGTTTCAAGCAGCAACCTTAACCCGAGTTTCGGCGTG

ATTTGGCAGCCGCACGAACACTGGAGCTTCAGCGCAAGCCACAACTACGCCAGCCGCAGC

CCGCGCCTGTATGACGCGCTGCAAACCCACGGCAAACGCGGCATCATCTCGATTGCCGAC

GGCACCAAAGCCGAACGCGCGCGCAATACCGAAATCGGCTTCAA
